# Design, synthesis and modelling of photoreactive chemical probes for investigating target engagement of plasmepsin IX and X in *Plasmodium falciparum*†

**DOI:** 10.1039/d3cb00109a

**Published:** 2023-11-13

**Authors:** Monika Lisauskaitė, Gemma L. Nixon, Christopher M. Woodley, Neil G. Berry, Andy Coninckx, L. Charlie Qie, Suet C. Leung, Donatella Taramelli, Nicoletta Basilico, Silvia Parapini, Stephen A. Ward, Oscar Vadas, Dominique Soldati-Favre, W. David Hong, Paul M. O'Neill

**Affiliations:** a Department of Chemistry, University of Liverpool Liverpool L69 7ZD UK pmoneill@liverpool.ac.uk; b Dipartimento di Scienze Farmacologiche e Biomolecolari (DISFEB), Università degli Studi di Milano 20133 Milano Italy; c Dipartimento di Scienze Biomediche, Chirurgiche e Odontoiatriche, Università degli Studi di Milano 20133 Milano Italy; d Dipartimento di Scienze Biomediche per la Salute, Università degli Studi di Milano 20133 Milano Italy; e Affiliated to Centro Interuniversitario di Ricerche sulla Malaria/Italian Malaria Network (CIRM-IMN), Università degli Studi di Camerino Italy; f Department of Tropical Disease Biology, Liverpool School of Tropical Medicine Liverpool L3 5QA UK; g Department of Microbiology and Molecular Medicine, Faculty of Medicine, CMU, 1 rue Michel-Servet CH-1211 Genève 4 Switzerland

## Abstract

The emergence of *Plasmodium* parasite resistance to current front-line antimalarial treatments poses a serious threat to global malaria control and highlights the necessity for the development of therapeutics with novel targets and mechanisms of action. Plasmepsins IX and X (PMIX/PMX) have been recognised as highly promising targets in *Plasmodium* due to their contribution to parasite's pathogenicity. Recent research has demonstrated that dual PMIX/PMX inhibition results in the impairment of multiple parasite's life cycle stages, which is an important feature in drug resistance prevention. Herein we report novel hydroxyethylamine photoaffinity labelling (PAL) probes, designed for PMIX/PMX target engagement and proteomics experiments in *Plasmodium* parasites. The prepared probes have both a photoreactive group (diazirine or benzophenone) for covalent attachment to target proteins, and a terminal alkyne handle allowing their use in bioorthogonal ligation. One of the synthesised benzophenone probes was shown to be highly promising as demonstrated by its outstanding antimalarial potency (IC_50_ = 15 nM *versus* D10 *P. falciparum*) and its inhibitory effect against *Pf*PMX in an enzymatic assay. Molecular docking and molecular dynamics studies show that the inclusion of the benzophenone and alkyne handle does not alter the binding mode compared to the parent compound. The photoaffinity probe can be used in future chemical proteomics studies to allow hydroxyethylamine drug scaffold target identification and validation in *Plasmodium*. We expect our findings to act as a tool for future investigations on PMIX/PMX inhibition in antimalarial drug discovery.

## Abstract 2

We report the synthesis and biological profiling of photoreactive chemical probes based on an established plasmepsin X inhibitor scaffold. Maintained antimalarial and recombinant enzyme activity support their use in future chemical proteomic studies.

## Graphical abstract

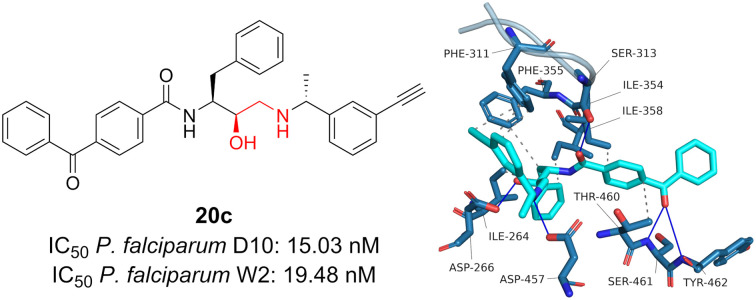

## Introduction

Malaria is a life-threatening infectious disease caused by protozoan parasites of *Plasmodium* genus. It has been part of human history for thousands of years, yet its threat to the health and wellbeing of people worldwide remains of great concern today.^1–3^ In 2021 alone, there were 247 million estimated malaria cases and 619 000 associated deaths – 76% of which occurred in children under the age of five.^4^ Currently, the front-line antimalarial treatments are artemisinin combination therapies (ACTs). However, the extensive use of ACTs has resulted in *Plasmodium* resistance against artemisinin and its derivatives.^5–11^ This poses a serious threat to global malaria control and aspirations of disease elimination. Due to the life-threatening nature of the disease, the resistance emergence to current treatments is extremely daunting and calls for the development of novel antimalarial drugs with unexplored mechanisms of action.

Since artemisinin resistance was first reported in 2008, the scientific community responded with a rise in antimalarial high-throughput screening campaigns both in academia and pharmaceutical companies.^5,12–15^ This resulted in identification of thousands of starting points for hit to lead antimalarial drug optimisation. Additionally, the importance of their target identification has been recognised to allow the monitoring of potential resistance acquisition mechanisms and development. One such protein group – aspartic proteases – has been recognised as a promising target in *Plasmodium falciparum* due to its contribution to parasite's pathogenicity.^16–18^ Overall, the parasite has ten aspartic proteases named plasmepsins I–X (PMI-X) that are involved in important biological processes.^16^ Plasmepsins IX and X are of a particular interest due to their necessity for parasite survival and targeting of the symptomatic intraerythrocytic life cycle stage (erythrocyte invasion and egress).^19–21^ PMIX is important in red blood cell (RBC) invasion and parasitophorous vacuolar membrane (PVM) set up, while PMX is required for both RBC invasion and egress.^16,19^ PMX inhibition can trap the parasite within PVM and RBCs.^20^ In addition to this, PMX is also expressed in the sexual stage of *P. falciparum* where its inhibition is known to affect the parasite egress from gametocytes and the invasion of vector's midgut.^20^ PMX inhibition also interferes with parasite's progression from the liver to RBCs either through merosome formation blocking or effect on their function.^20^

**Fig. 1:**
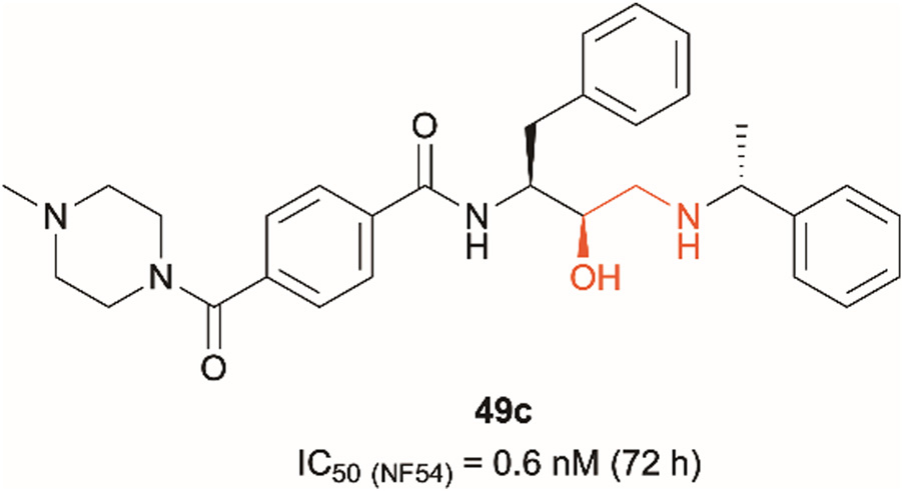
Hydroxyethylamine (red) containing PMIX/PMX inhibitor 49c and its reported antimalarial activity against chloroquine-resistant *Plasmodium* strain NF54 after 72 hours of treatment (activity data measured in house *versus* the D10 and W2 strains of *Plasmodium falciparum* are listed in Table 1 (*vide infra*)).^20,25^

Dual PMIX/PMX inhibition is considered a promising approach in antimalarial drug discovery due to its effect on blood stage *Plasmodium* (PMIX/PMX) as well as sexual and liver stage parasite (PMX). Such dual target inhibition can result in a lower risk of drug resistance acquisition. Therefore, development of such therapeutics is being explored and has been reported in the literature.^20,22^ Hydroxyethylamine derivatives have previously been studied as potential antimalarials targeting plasmepsins due to their pharmacophore structural similarity to aspartic protease substrates.^23–26^ The effect that the hydroxylamine scaffold has on plasmepsin function has been demonstrated by compound 49c which was shown to prevent intraerythrocytic parasite's release from the PVM and host RBCs, suggesting that the drug acts as a PMX inhibitor (Fig. 1).^20,25^ It was also capable of inhibiting erythrocyte invasion after 5 hours of treatment, suggesting that it might be acting as a dual PMIX/PMX inhibitor. 49c was further tested in a mouse model infected with *P. berghei*, which showed both parasite clearance in infected mice and interreference with disease transmission – the drug caused blocking of oocyst formation in Anopheles mosquito's midgut following its blood meal. Here we report the development of novel ‘clickable’ photoaffinity labelling (PAL) probes based on the hydroxylamine scaffold, that will enable bioorthogonal ligation and target protein identification within living parasites. This approach will provide solid *in vitro* proof of principle for selective engagement of plasmepsin IX/X over other know plasmepsin targets.^21^

## Results and discussion

### Design and synthesis of diazirine photoaffinity probes

The development of ‘clickable’ PAL ligands for drug target identification remains challenging as two additional motifs (PAL warhead and ligation handle) have to be incorporated/merged within the ligand through an extensive knowledge of SAR. For this reason, the design of our hydroxyethylamine photoaffinity probes was focused on three key structural aspects. Firstly, in order to avoid compromising the scaffold's antimalarial activity, no chemical modifications were performed on the hydroxyethylamine moiety. The *S*,*R* chirality of this class of compounds is known to be necessary for maintaining good potency and was not altered during the probe development process.^25^ Secondly, the attachment site of the photoreactive groups had to be placed either on the left-hand side or the right-hand side of the molecule based on our understanding of the structure–activity relationship (SAR). As noted, in order for the probes to be compatible with bioorthogonal ligation, incorporation of a terminal alkyne handle in the structure was also a key design element.

**Fig. 2:**
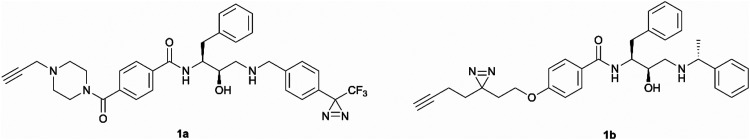
Design of aromatic (1a) and aliphatic (1b) diazirine photoaffinity probes prioritised for synthesis and biological activity evaluation in *P. falciparum*.

Photoaffinity probes 1a and 1b were specifically designed to incorporate diazirine moieties into their structures (Fig. 2). The photoreactive diazirine group was prioritised over other alternatives due to its small size, long excitation wavelengths, high photocrosslinking efficiency, relatively high thermal/chemical stability, and commercial availability.^27^ Both aromatic (1a) and aliphatic (1b) diazirines were chosen for investigation. In probe 1a, the alkyne handle was attached at the piperazine as structural changes on cyclic/tertiary amides appeared to be tolerated based on the inhibitor's SAR. The aromatic diazirine was incorporated into the right-hand side to mimic the benzyl substituent of 49c that was shown to tolerate both *para* and *meta* substitution with methoxy, fluoro and nitrile functionalities.^25^ Probe 1b utilised a diazirine linker with a minimalistic design, which was anticipated to be less bulky and increase the likelihood of avoiding potency loss.

**Scheme 1:**
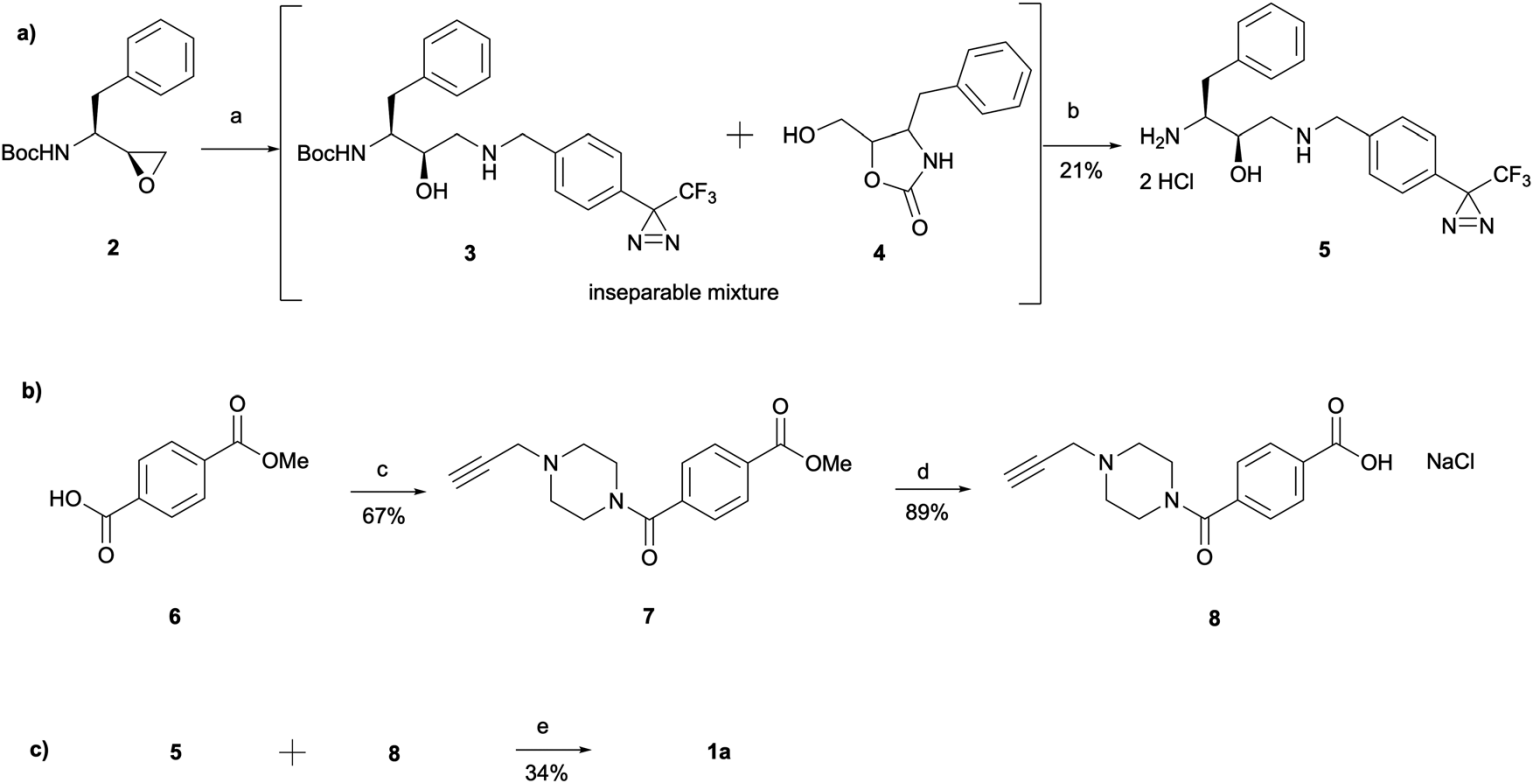
Synthesis of probe 1a consisting of three main parts: (a) amine synthesis, (b) carboxylic acid synthesis and (c) amide coupling between the two. *Reagents and conditions*: (a) (4-(3-(Trifluoromethyl)-3*H*-diazirin-3-yl)phenyl)methanamine, Et_3_N, MeOH, 60 °C, 6 h; (b) HCl 4 M in dioxane, DCM, r.t., o/n; (c) oxalyl chloride, DCM, DMF, r.t., 3 h, followed by 1-(prop-2-yn-1-yl)piperazine, THF, r.t., 1.5 h; (d) NaOH, MeOH, 60 °C, 3 h, followed by HCl; (e) HATU, 4-ethylmorpholine, DMSO, r.t., o/n.

The synthesis of 1a involved a three-part strategy encompassing the preparation of a diazirine-containing amine 5, the synthesis of a carboxylic acid 8 incorporating a terminal alkyne, and the amide coupling between the two compounds to yield the photoaffinity probe 1a (Scheme 1). Amine preparation began with ring-opening of epoxide 2 yielding an inseparable mixture of hydroxyethylamine moiety containing intermediate 3 and cyclic byproduct 4 (structure determined following amine deprotection). The crude mixture was subjected to an overnight HCl assisted Boc deprotection in DCM. By taking advantage of the polarity difference between amine 5 and byproduct 4, a separation method involving product precipitation was employed, which allowed successful isolation of the desired product 5 and facilitated the elucidation of the structure of the byproduct 4. Reports of *N*-Boc-epoxide cyclisation explaining the observed byproduct formation can be found in the literature where it has been shown that the cyclic intermediate can be obtained by a 5-*exo* ring-opening reaction of the epoxide.^28^ Since the purification of the Boc-protected intermediate 3 was found to be unnecessary for obtaining pure amine, the tandem approach of epoxide opening and deprotection was employed for the synthesis of all other probes that required this step (*vide infra*). It is worth noting that despite the formation of a polar byproduct being observed by TLC in other epoxide opening reactions, its isolation or further studies on conditions required to prevent this intramolecular byproduct formation were not performed as it did not show significant interference with the synthesis or purification of the HCl amine salts.

The synthesis of the carboxylic acid 8 began with an oxalyl chloride/DMF assisted amide coupling reaction between the 4-(methoxycarbonyl)benzoic acid 6 and 1-(prop-2-yn-1-yl)piperazine to allow the introduction of an alkyne handle into the probe structure. Intermediate 7 was then subjected to NaOH catalysed ester hydrolysis affording intermediate 8. Following the synthesis of both amine 5 and carboxylic acid 8, HATU activated amide coupling was performed to yield the desired photoaffinity probe 1a.

**Scheme 2:**
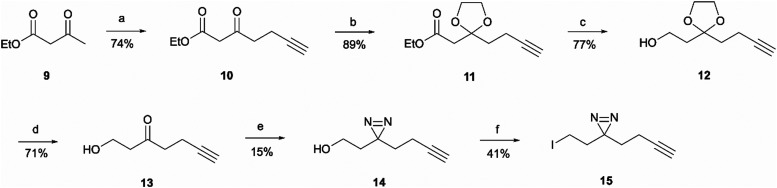
Synthesis of minimalistic terminal alkyne-containing diazirine linker. *Reagents and conditions*: (a) *n*-BuLi, DIPA, THF −78 °C for 15 min before addition of 9, then add and stir for 30 min at 0 °C; add propargyl bromide at 0 °C followed by r.t., o/n; (b) Ethylene glycol, triethyl orthoformate, TsOH·H_2_O, r.t., o/n; (c) LiAlH_4_, THF, 80 °C, 2 h; (d) TsOH·H_2_O, acetone/water (5 : 1), 75 °C, 6 h; (e) 7 M NH_3_ in MeOH, NH_2_OSO_3_, o/n followed by I_2_, Et_3_N, MeOH, 0 °C, 1 h; (f) I_2_, PPh_3_, imidazole, THF, r.t., 4 h.

For the synthesis of the aliphatic diazirine photoaffinity probe 1b, a minimalistic diazirine substituent first had to be prepared (Scheme 2). The synthetic route commenced with an alkyne handle incorporation to ethyl acetoacetate 9 yielding intermediate 10 which then underwent ketone protection to produce 11. Isolation of intermediates 10 and 11 was achieved in 74% and 89% yields, respectively, which was comparable to literature reports.^29,30^ This was then followed by ester reduction with LiAlH_4_ to give the hydroxy intermediate 12 which was deprotected under acidic conditions affording ketone 13. The ketone was then converted to diazirine 14 by treating it with 7 M ammonia in methanol and hydroxylamine-*O*-sulfonic acid. This results in the formation of diaziridine that can be further oxidised with iodine in the presence of triethylamine.^31^ The performed synthesis allowed diazirine 14 isolation in a 15% yield. It is worth noting that the use of 7 M ammonia in methanol solution normally results in lower yields but is more convenient and safe to handle, while higher yields of 40–80% are achievable when liquid ammonia is employed.^32–34^ The formed diazirine intermediate underwent alcohol conversion to iodide 15 that could be used in S_N_2 reactions as the electrophile.

**Scheme 3:**
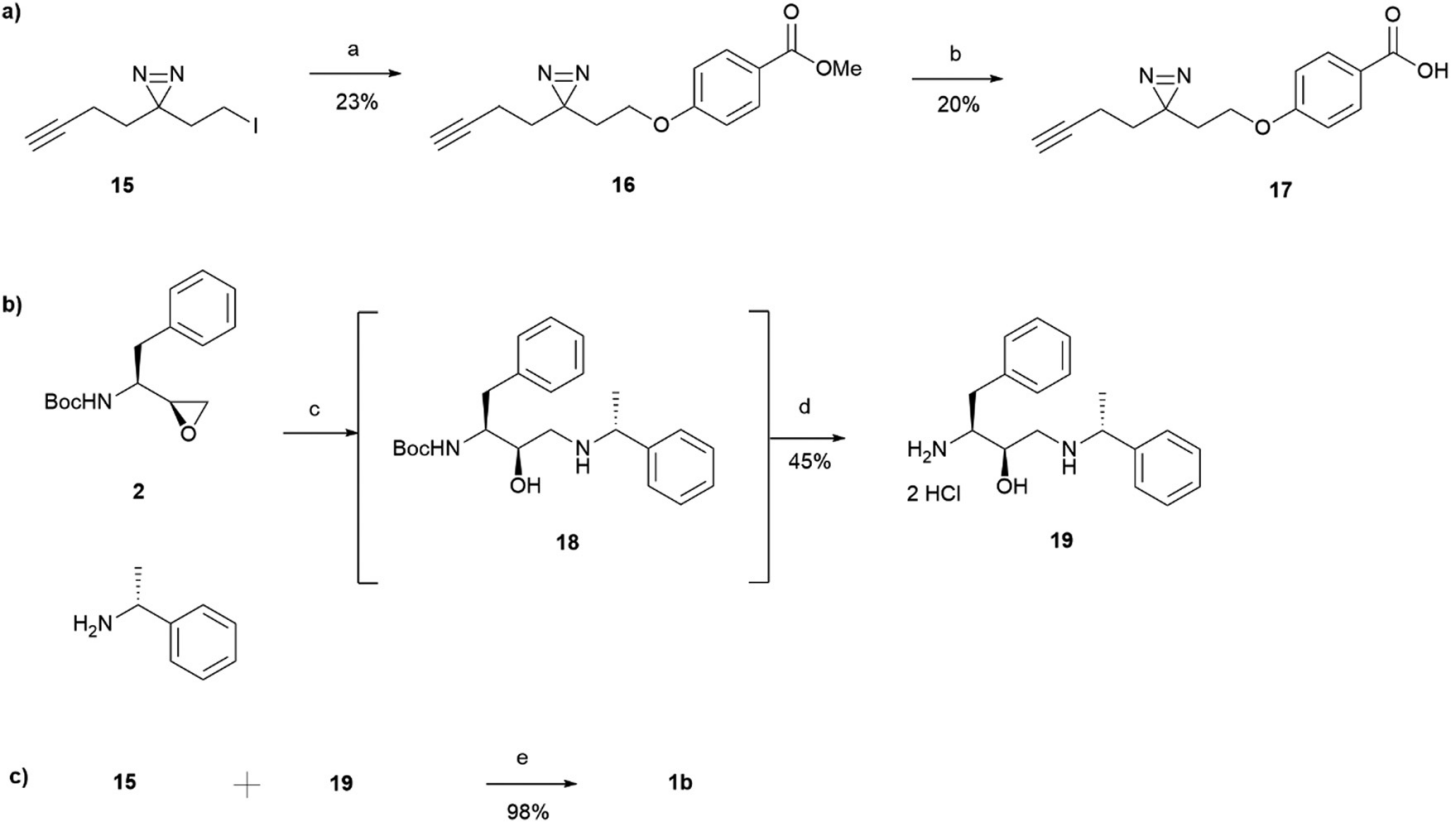
Synthesis of probe 1b consisting of three main parts: (a) carboxylic acid synthesis, (b) amine synthesis and (c) amide coupling between the two. *Reagents and conditions*: (a) Methyl 4-hydroxybenzoate, K_2_CO_3_, DMF, 60 °C, 48 h; (b) NaOH, MeOH, 50 °C for 3.5 h, then r.t. overnight and 50 °C for further 24 hours followed by HCl (c) (*R*)-1-phenylethan-1-amine, MeOH, 65 °C, o/n; (d) HCl 4 M in dioxane, DCM, r.t., o/n; (e) HATU, 4-ethylmorpholine, DMSO, r.t., o/n.

15 was incorporated into the main probe pharmacophore as outlined in Scheme 3. Similarly to the synthesis of 1a, the route for the preparation of photoaffinity probe 1b consisted of three main parts: left-hand side carboxylic acid synthesis, right-hand side amine synthesis, and amide coupling between the two. Synthesis of the carboxylic acid 17 began with an S_N_2 reaction between methyl 4-hydroxybenzoate and the prepared minimalistic linker 15, affording intermediate 16 in a 23% yield. Unfortunately, even after heating at 60 °C for 48 hours, the formation of ether 16 was still in an undesired low yield with a significant proportion of starting material remaining. The ester intermediate 16 was then hydrolysed with NaOH affording the left-hand side building block 17 in a 20% yield. The acid 17 was precipitated using HCl and was used in the next step without further purification. Synthesis of the right-hand side amine building block followed a similar procedure as that reported for the synthesis of amine 5. In a similar fashion to intermediate 3, Boc-protected intermediate 18 could not be purified due to the formation of byproduct of almost identical polarity. Crude 18 was therefore used in the HCl mediated Boc deprotection to afford amine 19 in a 45% yield. HATU activated amide coupling between carboxylic acid 17 and amine 19 afforded the desired photoaffinity probe 1b in a 98% yield.

### Design and synthesis of benzophenone photoaffinity probes

**Fig. 3:**
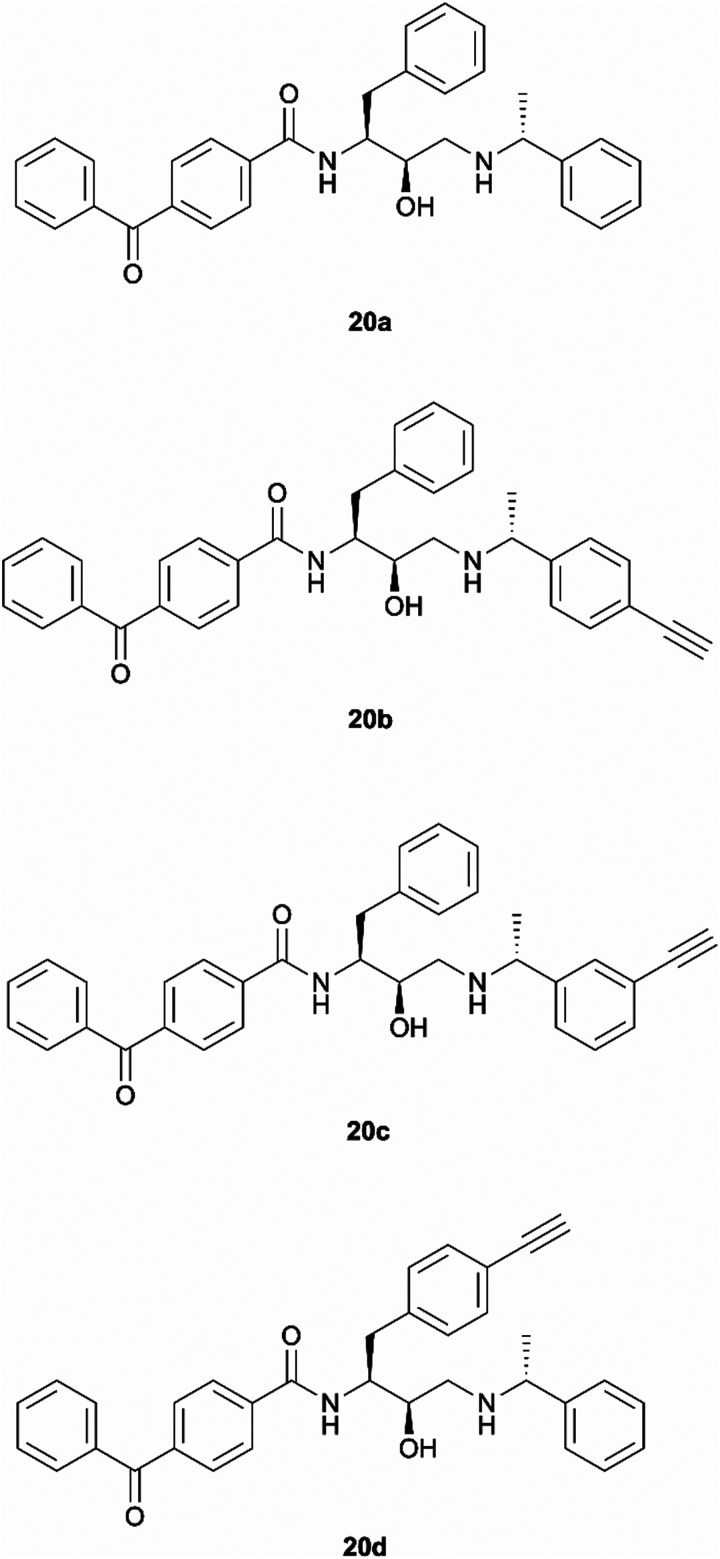
Design of benzophenone probes 20a–20d for synthesis and biological activity evaluation in *P. falciparum*.

Benzophenone was also investigated as an alternative photoaffinity group to the diazirine to probe the effect of the different substitutions on antimalarial activity. The photoreactive group's compatibility with synthetic strategies allowing aromatic ring incorporation into the drug core structure could be exploited on our scaffold. The more desired position for benzophenone incorporation was the left-hand side of the molecule where *N*-methyl piperazinyl in 49c was replaced by an unsubstituted phenyl group. With this in mind, probes 20a–20d were designed (Fig. 3). Probe 20a was expected to allow the evaluation of the effect that incorporation of benzophenone has on the scaffold's potency, while compounds 20b–20d would explore the most suitable position for alkyne ligation handle attachment.

**Scheme 4:**
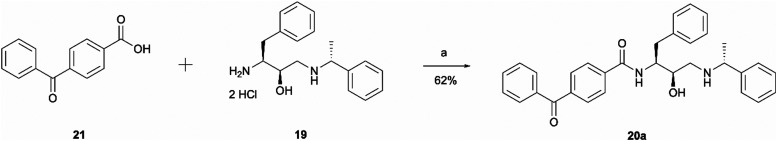
Synthesis of benzophenone photoaffinity probe 20a. *Reagents and conditions*: (a) HATU, 4-ethylmorpholine, DMSO, r.t., o/n.

The synthesis of probe 20a was a simple one step amide coupling reaction between acid 21 and the previously synthesised amine hydrochloride salt 19 (Scheme 4). The desired photoaffinity probe was isolated in a 62% yield.

**Scheme 5:**
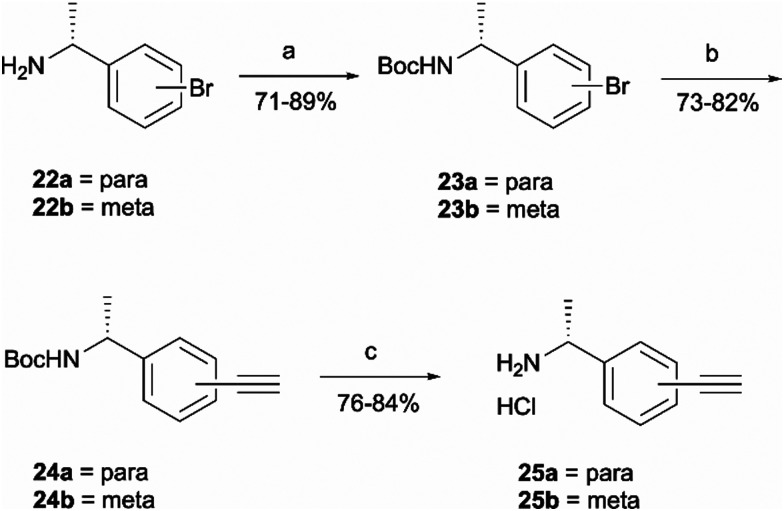
Synthesis of right-hand side amines containing an alkyne handle. *Reagents and conditions*: (a) Boc_2_O, Et_3_N, DCM, r.t., 4 h (b) trimethylsilylacetylene, Pd(PPh_3_)_2_Cl_2_, CuI, Et_3_N, 70 °C, o/n, then concentrate and treat with K_2_CO_3_, MeOH, r.t., 3 h; (c) HCl 4 M in dioxane, MeOH, r.t., o/n.

Probes 20b and 20c were proposed as the SAR suggests that *meta* and *para* substitution at the right-hand side ring should be tolerated.^25^ This was therefore identified as the most promising approach for alkyne handle attachment. In order to prepare the proposed photoaffinity probes, right-hand side amines containing an alkyne handle had to be synthesised (Scheme 5) first. The synthesis began with Boc protection of amine 22a/22b to yield intermediates 23a/23b in reasonably high yields of 71% and 89%, respectively. 23a/23b were then subjected to Sonogashira coupling to introduce the alkyne handles. The procedure consisted of two main parts: trimethylsilylacetylene attachment to the aromatic ring through Pd/Cu catalysis, followed by trimethylsilyl group removal with base to produce the final terminal alkyne intermediates 24a/24b in 73% and 82% yields. The resulting amine intermediates were then deprotected to yield their hydrochloride salts 25a/25b ready for coupling to the main drug pharmacophore.

**Scheme 6:**
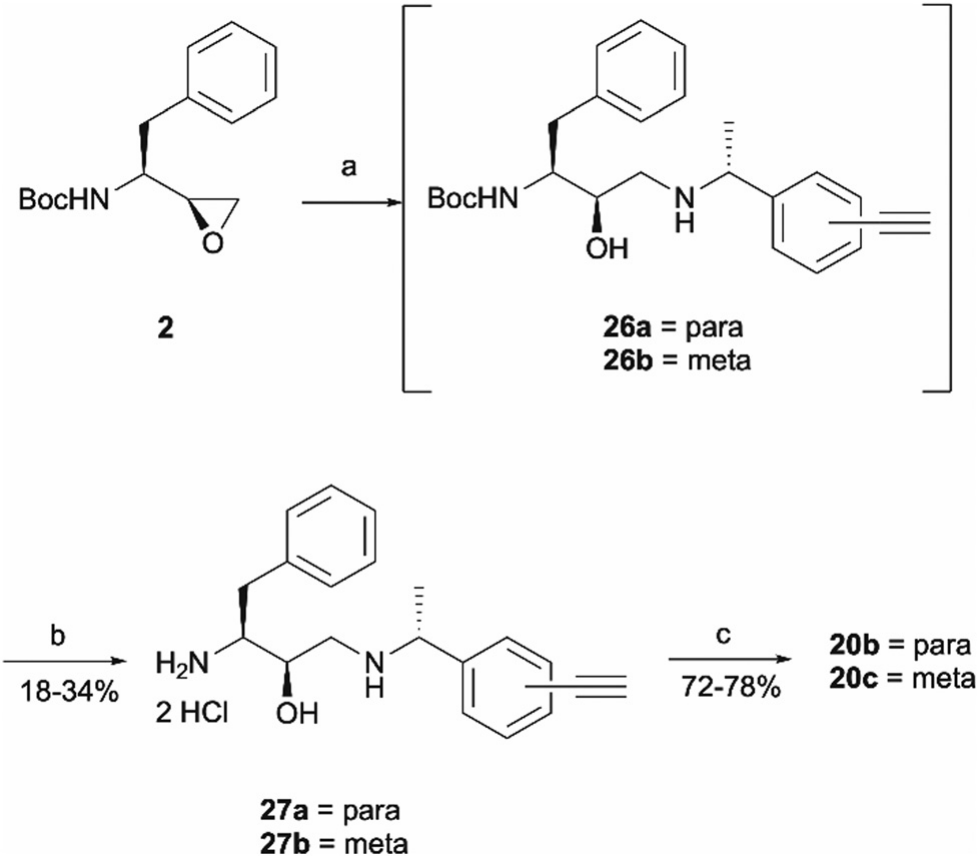
Synthesis of probes 20b and 20c. *Reagents and conditions*: (a) Amines 25a/25b, Et_3_N, MeOH, 65 °C, o/n; (b) HCl 4 M in dioxane, DCM, r.t., o/n; (c) 4-benzoylbenzoic acid 21, HATU, 4-ethylmorpholine, DMSO, r.t., o/n.

Once the right-hand side amines 25a/25b were synthesised, they were used in epoxide opening reactions, which similarly to those described before, resulted in the formation of crude intermediates 26a/26b (Scheme 6). They were deprotected to afford the amine building blocks 27a/27b in 18% and 34% yields, respectively. The prepared amines were then coupled with 4-benzoylbenzoic acid 21 to give the photoaffinity probes 20b and 20c in 72% and 78% yields.

**Scheme 7:**
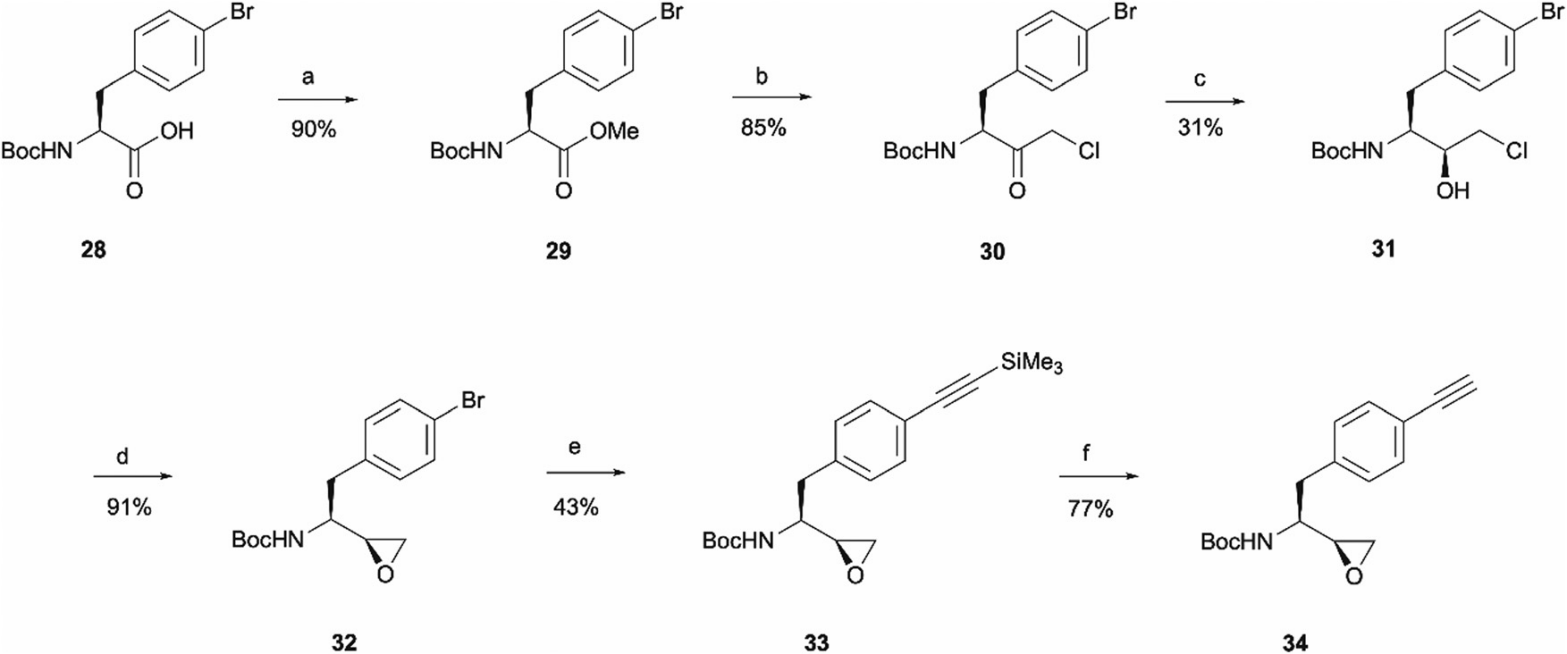
Epoxide synthesis. *Reagents and conditions*: (a) MeI, NaHCO_3_, DMF, r.t., o/n (b) ^*t*^BuMgCl, sodium chloroacetate, Et_3_N, THF, 0 °C to r.t., o/n, then aq. citric acid; (c) NaBH_4_, EtOH, r.t., 1 h, then H^+^ (d) KOH, EtOH, r.t., 1 h; (e) trimethylsilylacetylene, Pd(PPh_3_)_2_Cl_2_, CuI, Et_3_N, 70 °C; (f) TBAF, THF, r.t., 15 min.

The rationale behind the design of photoaffinity probe 20d was to examine the potential of the central benzene ring as a viable option for attaching an alkyne handle. In order to introduce the terminal alkyne into the probe's structure at the desired position, a new alkyne handle containing epoxide 34 had to be synthesised (Scheme 7). The synthetic route for the epoxide preparation began with an esterification reaction that converted the carboxylic acid starting material 28 into a methyl ester 29. The methyl ester was chosen instead of an ethyl ester or other alternatives as the subsequent chloroketone formation step was reported to work better on methyl esters in the literature.^35^ The intermediate was then used in a chloromethylation reaction to afford 30 in an 85% yield. It is worth noting that in this step, reagents sodium chloroacetate and butylmagnesium chloride are used in excess for the deprotonation of the BocNH proton, avoiding enolisation and consequent racemisation of the chiral centre.^35^ According to work performed by M. J Zacuto *et al.*, it is important that in this step the reaction mixture is added to the citric acid solution and not the other way around as it prevents Mg salt precipitation which would complicate the work-up procedure.^36^

Once the chloromethyl functionality was introduced into the structure, the ketone in compound 30 had to be reduced to the corresponding alcohol 31 to allow the intramolecular S_N_2 reaction for epoxide formation. The reduction allowed the isolation of 31 in a 31% yield and 98 : 2 d.r. The erythro (*S*,*S*) and threo (*S*,*R*) diastereomers of leucine, phenylalanine, alanine and valine derived Boc-amino epoxides can be determined through NMR analysis.^37^ Ketone reduction yielding the hydroxy erythro intermediate is known to show an NH peak at 4.6 ppm in CDCl_3_, while in the threo intermediate the same peak appears at 4.7 ppm. The diastereomeric purity of 31 was consistent in both HPLC and NMR analysis. The observed diastereoselectivity has been thoroughly discussed by O. K. Karjalainen and A. M. P. Koskinen who used a Felkin-Ahn model to propose mechanistic paths that may take place during the ketone reduction depending on the reducing agent used.^38^ In our case, reasonably selective reduction was achieved using NaBH_4_ in the formation of intermediate 31.^38^ The correct stereochemistry of the hydroxy intermediate was further confirmed by its conversion to the erythro epoxide 32 that showed the absence of any peaks at 2.99 ppm in the ^1^H NMR spectra, which would correspond to the threo diastereomer methine peak. In the erythro isomer the same peak would appear at 2.84 ppm which agrees with the multiplet at 2.86–2.64 ppm.^37^ Following epoxide 32 synthesis, trimethylsilylacetylene was introduced through Sonogashira coupling. The trimethylsilyl group in intermediate 33 was then removed to obtain the terminal alkyne handle which would make the probe suitable for bioorthogonal ligation. This was achieved employing a 15 min reaction with TBAF that allowed alkyne handle-containing compound 34 isolation in a 77% yield.

**Scheme 8:**
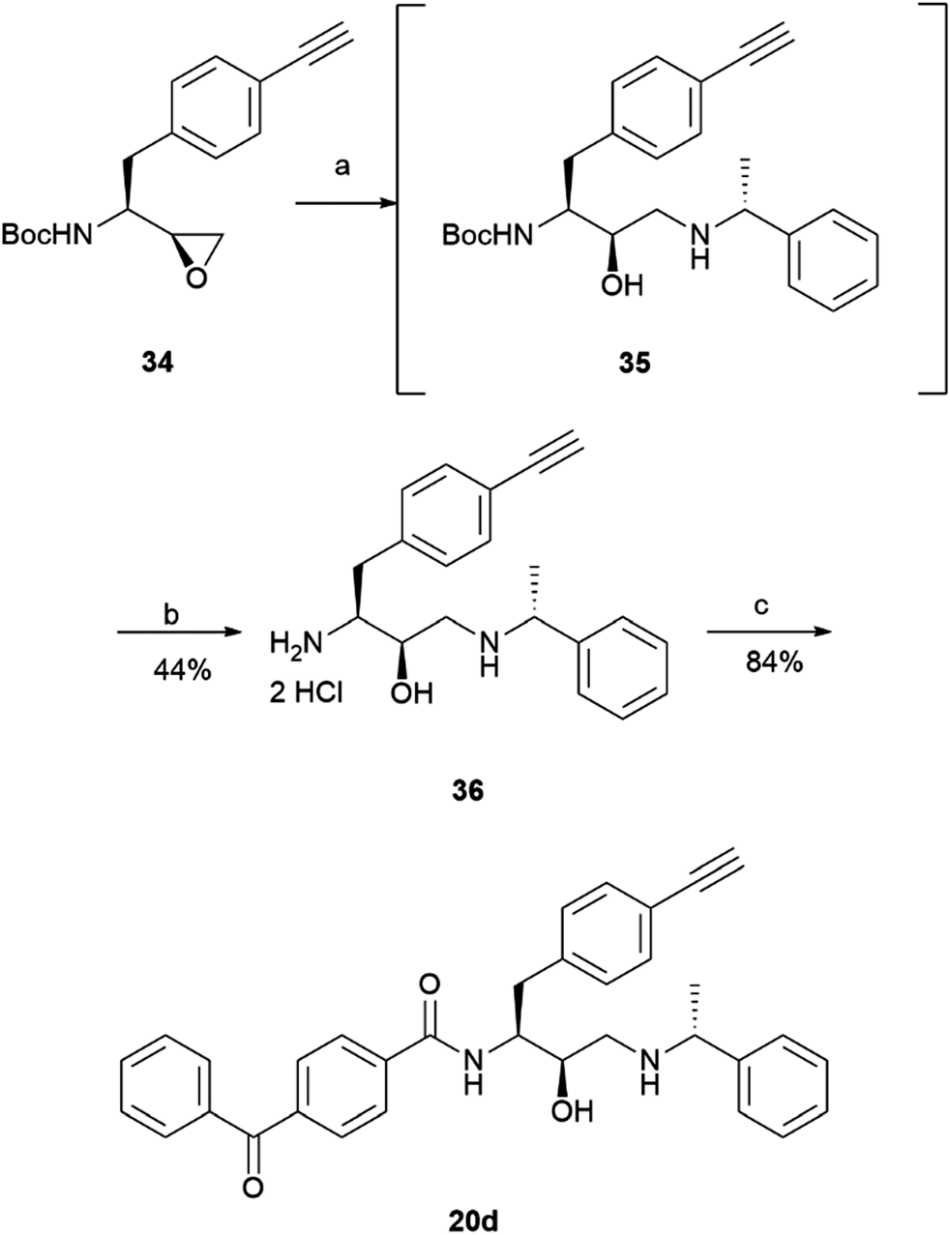
Synthesis of probe 20d. *Reagents and conditions*: (a) 1-phenylethan-1-amine, MeOH, 65 °C, o/n; (b) HCl 4 M in dioxane, DCM, r.t., o/n; (c) 4-benzoylbenzoic acid 21, HATU, 4-ethylmorpholine, DMSO, r.t., o/n.

The prepared intermediate 34 was then used in an epoxide opening reaction, which similarly to those described in the earlier pages, resulted in a formation of crude intermediate 35 (Scheme 8). The crude Boc-protected amine was then subjected to HCl assisted deprotection affording intermediate 36 in a 44% yield. The amine salt was used in a HATU activated amide coupling reaction with the photosensitive 4-benzoylbenzoic acid 21 to afford the desired benzophenone photoaffinity probe 20d in an 84% yield.

### Antimalarial activity of photoreactive chemical probes

All the prepared photoreactive chemical probes were tested against *P. falciparum in vitro* to determine their biological activity (Table 1). Antimalarial activity was determined against chloroquine sensitive (D10) and chloroquine resistant (W2) strains by measuring parasite viability after 72 hours of incubation with the probes. The potency against D10 and W2 strains was comparable for all probes. Except for the aromatic diazirine probe 1a, all other photoaffinity probes exhibited sub-micromolar activity against both *P. falciparum* strains. In general, benzophenone probes were more potent than diazirine probes. Good potency of probe 20a demonstrated that incorporation of the photoreactive benzophenone group has lower impact to the scaffold's antimalarial activity than the incorporation of diazirine group. *Meta*-substituted probe 20c showed best activity out of all probes with an IC_50_ < 20 nM against both D10 and W2 strains of parasites, while the remaining benzophenone probes 20b and 20d exhibited 200–300 nM (IC_50_) activity.

**Table 1:** Antimalarial activity of the photoreactive chemical probes measured against *P. falciparum* after 72 hours of incubation. D10 is a chloroquine sensitive parasite strain, while W2 is a chloroquine resistant strain. Chloroquine (IC_50_ = 15.53 ± 4.56 nM (D10) and 236.84 ± 81.56 (W2)) was used as a positive control in the assay (*n* = 3)

Probe	IC_50_ D10 (nM)	IC_50_ W2 (nM)
1a	1021.51 ± 240.04	1245.03 ± 429.57
1b	207.78 ± 86.98	195.96 ± 79.02
20a	18.29 ± 5.06	23.34 ± 3.50
20b	270.96 ± 96.61	299.58 ± 59.45
20c	15.03 ± 5.58	19.48 ± 6.54
20d	165.69 ± 45.92	204.33 ± 51.35
49c	0.77 ± 0.32	0.91 ± 0.28

### Enzymatic activity of selected photoreactive chemical probes against *Pf*PMX

**Fig. 4:**
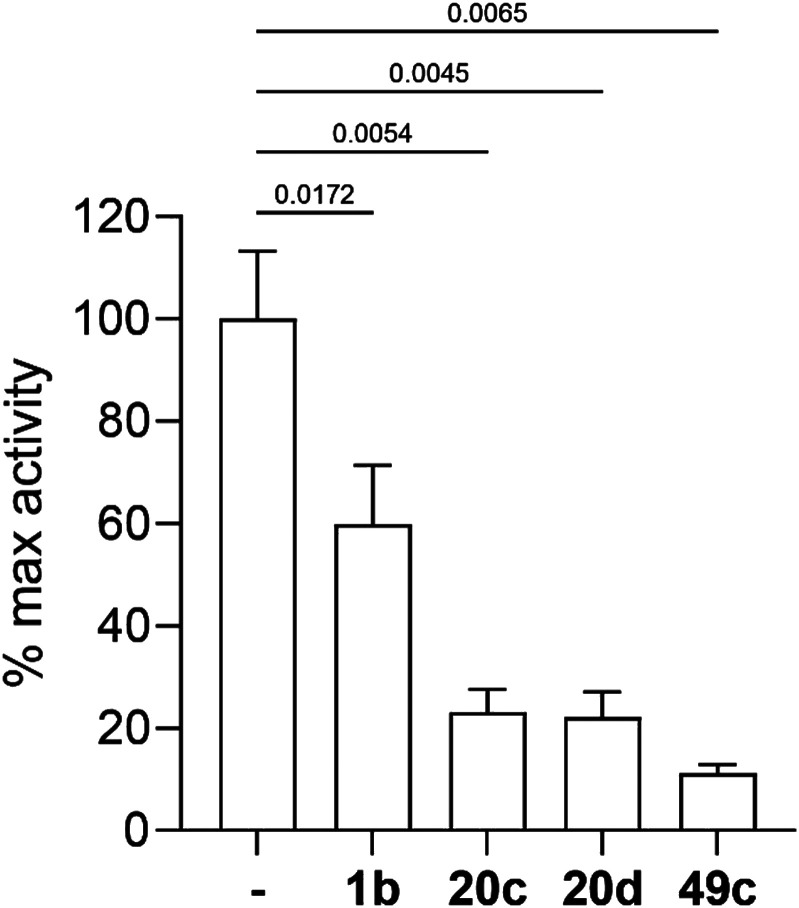
Inhibition of recombinant *Pf*PMX activity by selected photoreactive chemical probes, tested on *Pf*SUB1 peptide substrate. Results shown as mean + SD (*n* = 3) normalised to the activity in absence of inhibitor (DMSO control). *P*-Values calculated by unpaired *t*-test.

Three selected photoreactive chemical probes, 1b, 20c and 20d, were tested in a biochemical *in vitro* assay (peptide substrate cleavage) against recombinant *Plasmodium falciparum* PMX (*Pf*PMX) with 49c as the positive control (Fig. 4).^39^ In this enzymatic assay, all three probes showed significant inhibition of *Pf*PMX activity when used at 1 μM. The two probes with the photoreactive benzophenone group, 20c and 20d showed similar inhibitory effect as the positive control 49c while the diazirine incorporated probe, 1b, showed slightly reduced inhibitory potency. These *in vitro* data show that the antimalarial activity of these probes was at least partially due to their capability of inhibiting *Pf*PMX, one of the intended targets of 49c.

### Molecular docking and molecular dynamics studies

**Fig. 5:**
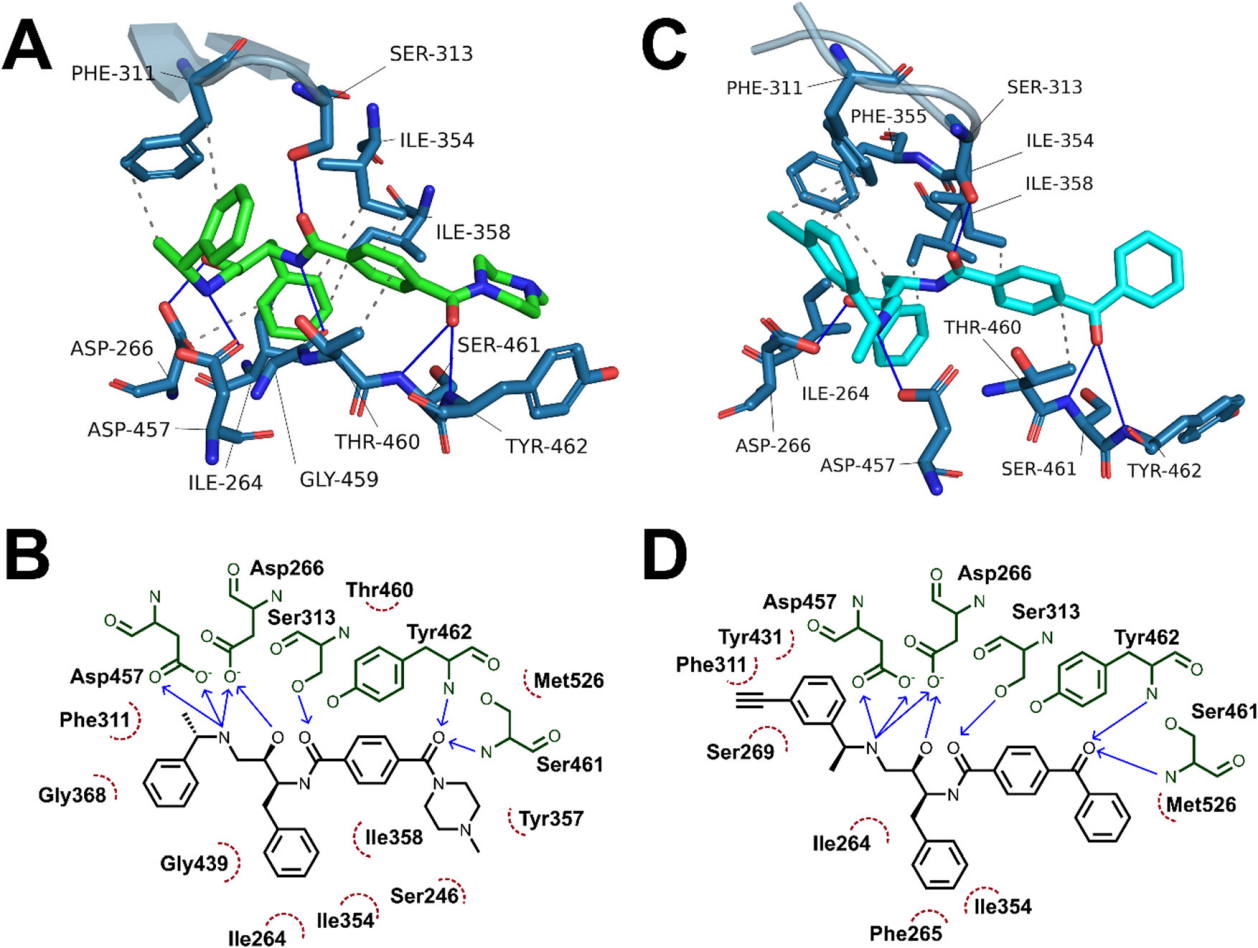
(A) and (C) Interaction analyses of end-state binding modes of 49c (A, green sticks) and 20c (cyan sticks) in PMX. Binding site residues are shown as blue sticks, the flap region is shown as a blue cartoon representation, hydrogen bonds are shown as blue lines, hydrophobic interactions are shown as dotted grey lines. Interactions were detected using the protein ligand interaction profiler tool. (B) and (D) 2D representations of non-covalent interactions between 49c (A) and 20c (B) with binding site residues. Binding site residues are shown in green, hydrophobic interacting residues are depicted by red, dotted arcs, hydrogen bond donors are depicted as blue arrows point from donor to acceptor.

Having demonstrated the antimalarial activity and the enzymatic activity against *Pf*PMX of the probes, molecular docking and molecular dynamics (MD) simulations were used to determine whether the most active probe, 20c could form sufficiently similar binding interactions to the parent compound 49c. To generate suitable protein structures to dock the probe into, molecular dynamics studies carried out by Kesari *et al.* were replicated using 49c docked into an experimental crystal structure of PMX co-crystallised with small molecule inhibitor WM382.^40,41^ Triplicate 100 ns MD production runs were carried out and used to calculate molecular mechanics/Poisson-Boltzman surface area (MM/PBSA) binding free energy for the final 20 ns of the simulation and to determine a lowest energy binding mode.^42,43^ In agreement with Kesari *et al.*, 49c formed stable H-bonding interactions with Asp266 and Asp457 of the catalytic dyad (Fig. 5A–B), while crucially stabilising the flap region by a hydrogen bond between the central amide and Ser313 in our MD simulation.^40,44^ Decomposition of these interactions’ contribution to binding free energy suggests that the interaction between the charged amine and the deprotonated aspartate residues are the greatest contributing interaction to binding energy (Fig. S1, ESI†). Additional interactions detected over the course of the simulation included hydrogen bonds to Ser461 and Tyr462 and detected hydrophobic interactions with Ile354, Phe311, Thr460 and Tyr462.

The probes synthesised in this work were initially docked into a representative structure from the lowest energy MD run of 49c (interaction analyses of all docked probes is provided in the ESI†). The highest ranking pose of 20c was prepared for MD simulation using the same method and settings as 49c. Production MD runs of 100 ns were ran in triplicate and analysed by MM/PBSA and clustering methods. Two of the three MD simulations of 20c, runs 1 and 3, converged to similar MM/PBSA predicted binding energies, while the binding mode generated in run 2 had weaker binding energy.

The average MM/PBSA binding energy for 20c run 1 was similar to the value obtained for 49c (−426.2 ± 26.2 *versus* −442.727 ± 16.8 kJ mol^−1^) with greatest contribution through the catalytic dyad, consistent with the comparable *in vitro* antimalarial activity. Similar to 49c, 20c likely forms stable hydrogen bonding interactions between ligand and the catalytic aspartate residues as well as the key flap-stabilising interaction between Ser313 and the central amide carbonyl (Fig. 5C–D). Additional stable hydrogen bonding interactions with Ser461 and Tyr462, as well as a network of hydrophobic interactions were also detected in the same MD run using 20c. The predicted binding energy of 20c run 3 was also similar to 49c (−429.4 ± 19.3 kJ mol^−1^) and maintained stable interactions with both aspartate residues and Ser313, however the terminal ring of the benzophenone bound further into the hydrophobic pocket defined by Tyr462. This resulted in hydrogen bonds between Ser461 and Tyr462 and the benzophenone carbonyl being lost in lieu of greater hydrophobic interactions, as evidenced by greater van der Waals contribution to binding free energy (Fig. S2 and Table S1, ESI†). The left-hand-side benzylamine group in 20c was observed to adopt an altered conformation compared to 49c – where in the predicted binding mode of 49c the methyl group points towards Phe311, in 20c the alkyne handle points towards Phe311 and the methyl group points towards the protein surface. This can be rationalised by the formation of stabilising hydrophobic interactions between the alkyne and the phenyl ring of Phe311 (Fig. 5C–D) – this is supported by the greater contribution of Phe311 to the calculated binding free energy of 20c*versus*49c.

A detailed summary of detected interactions and details of MM/PBSA analysis are provided in the ESI† (Tables S1 and S2). These molecular docking and molecular dynamics studies support that the *in vitro* activity of 20c likely occurs by a similar mechanism of action to 49c and suggest that 20c may be used as a chemical probe in target engagement and elucidation studies for antimalarial compounds related to 49c.

## Conclusions

In this study, we present the development of novel aspartic acid protease photoaffinity chemical probes containing photoreactive groups and a terminal alkyne moiety for bioorthogonal ligation in biological systems. By drawing on our understanding of the antimalarial SAR, we have managed to achieve the incorporation of photoreactive label and ligation handle within the parent scaffold whilst maintaining nanomolar drug potency. The incorporation of photoreactive groups is expected to allow covalent probe attachment to near proximity amino acid residues in the targeted proteins, while the inclusion of the terminal alkyne group will enable efficient and selective isolation of proteins under investigation through ‘click’ chemistry. Through this PAL based chemical proteomics approach, we will be able to provide a quantitative assessment of the degree of in-parasite plasmepsin labelling and determination of drug selectivity against plasmepsins IX and X over the other variants.^21^ Among the synthesised probes, 20c showed the most promising results in terms of antimalarial activity (IC_50_ < 20 nM) and proof-of-concept enzymatic inhibitory activity against one of the intended targets, *Pf*PMX. Subsequently, molecular docking and molecular dynamics studies in PMX demonstrated that the inclusion of the photoreactive group and terminal alkyne handle did not significantly alter the binding mode of the probe's scaffold in comparison to the parent 49c, further validating its potential.

## Author contributions

The project was conceptualised by ML, GN, SAW, WDH, CW, NBe, AC, LCQ, DT, SP and PMO’N. It was supervised by PMO’N, GN, WDH and NBe. The manuscript was written by ML and CW, with input from PMO’N, GN, SCL, WDH and NB. ML performed photoaffinity probe design, their synthesis and associated data analysis. CW performed molecular docking and molecular dynamic studies. DT, NBa and SP performed the biological studies. OV and DSF optimised and performed the *in vitro* plasmepsin X inhibition assays. All authors have read and approved the manuscript.

## Conflicts of interest

There are no conflicts to declare.

## Supplementary Material

CB-005-D3CB00109A-s001
