# Supplementary material for: Design, synthesis and modelling of photoreactive chemical probes for investigating target engagement of plasmepsin IX and X in Plasmodium falciparum
Source: RSC Chem Biol. 2023 Nov 13;5(1):19–29. doi: 10.1039/d3cb00109a (PMC10763550; doi:10.1039/d3cb00109a)
Supplement: CB-005-D3CB00109A-s001 [file CB-005-D3CB00109A-s001.pdf]

Electronic Supplementary Material (ESI) for RSC Chemical Biology.
This journal is © The Royal Society of Chemistry 2023

Supplementary Information

Design, synthesis and modelling of photoreactive chemical probes for investigating target engagement of plasmepsin IX and X in *Plasmodium falciparum*

Monika Lisauskaitė,[a] Gemma Nixon,[a] Christopher Woodley,[a] Neil Berry,[a] Andy Coninckx,[a] L. Charlie Qie,[a] Suet C. Leung,[a] Donatella Taramelli,[b,e] Nicoletta Basilico,[c,e] Silvia Parapini, [d,e] Steve A. Ward,[f] Oscar Vadas,[g] Dominique Soldati-Favre,[g] W. David Hong,[a] and Paul M. O'Neill*[a]

[a] Department of Chemistry, University of Liverpool, Liverpool, L69 7ZD, UK

[b] Dipartimento di Scienze Farmacologiche e Biomolecolari (DISFEB), Università degli Studi di Milano, 20133 Milano, Italy

[c] Dipartimento di Scienze Biomediche, Chirurgiche e Odontoiatriche, Università degli Studi di Milano, 20133 Milano, Italy

[d] Dipartimento di Scienze Biomediche per la Salute, Università degli Studi di Milano, 20133 Milano, Italy

[e] Affiliated to Centro Interuniversitario di Ricerche sulla Malaria/Italian Malaria Network (CIRM-IMN), Università degli Studi di Camerino, Italy

[f] Department of Tropical Disease Biology, Liverpool School of Tropical Medicine, Liverpool, L3 5QA, UK

[g] Department of Microbiology and Molecular Medicine, Faculty of Medicine, CMU, 1 rue Michel-Servet, CH-1211 Genève 4, Switzerland

Table of Contents

1 Synthetic Chemistry 3

1.1 General Remarks 3

1.2 General Procedures 3

1.3 Synthesis 5

1.4 HPLC Data 19

2 Biological Details 23

2.1. Antimalarial Activity Assay 23

2.2. PfPMX Peptide Cleavage Assay 23

3 Computational Details 23

3.1 Molecular Docking 23

3.2 Molecular Dynamics Simulations 23

4. Supplementary Notes 24

5. Supplementary Figures 25

6. Supplementary Tables 33

7 References 35

1 Synthetic Chemistry

1.1 General Remarks

Air- and moisture-sensitive reactions were performed in flame-dried glassware sealed with rubber septa under nitrogen atmosphere. Anhydrous liquids and solutions were transferred using syringes. Teflon-coated magnetic stirrer bars were used to mix all reaction mixtures. Unless stated, all materials were purchased from commercial sources (Alfa Aesar, CarboSynth, TCI, BroadPharm, Fluorochem and Sigma-Aldrich) and used without any further treatment. High-boiling solvents were removed from the reaction crudes employing rotary evaporators connected with high-vacuum pumps. Flash column chromatography was performed on silica gel (Aldrich, 40−63 µm, 230-400 mesh), eluting with various solvent mixtures and using compressed air to apply pressure. Thin layer chromatography (TLC) was performed using aluminium backed silica plates (Merck, 0.25 mm, 60 F-254). Visualisation was achieved by UV fluorescence or using ninhydrin, $KMnO_4$ or *p*-anisaldehyde stains followed by gentle heating with a hot air gun. Infrared spectra were recorded on a PerkinElmer Spectrum™ 3 FT-IR spectrometer. Nuclear magnetic resonance (NMR) spectra were recorded on either Bruker AMX 400 (^{1}H, 400 MHz; ^{13}C, 101 MHz) or Bruker 500 (^{1}H, 500 MHz; ^{13}C, 126 MHz) spectrometers. Chemical shifts (δ) are reported as parts per million (ppm) relative to residual solvent peak. Peaks are described as singlets (s), doublets (d), triplets (t), quartets (q), quintets (quint) multiplets (m) and broad (br.). When peak splitting appears as something it should not be in theory, it is referred to as apparent (app.). Coupling constants (J) are reported in Hertz (Hz). All assignments of NMR spectra were based either on 1D NMR alone, or 1D and 2D NMR (COSY, HSQC and HMBC). High resolution mass spectra (HRMS) were recorded on a Micromass LCT mass spectrometer by the University of Liverpool analytical services. HPLC analysis was performed using an Agilent 1200 system with the following conditions: ZORBAX Eclipse Plus C18 (4.6 mm x 100 mm, 3.5 µm) at 25 °C with 1.0 mL/min flow rate. Protocol: injection volume 5 µL; solvents – A) acetonitrile + 0.1% formic acid FA and B) water + 0.1% FA; method run time: 17 min, gradient: 2% A hold to 1 min, 2-98% A in 11 min, then hold at 98% A for 3 min, then from 98-2% A in 1 min, then holds at 2% A until run finishes.

1.2 General Procedures

General Procedure A: Epoxide Opening and Boc-Deprotection

Amine/amine salt (1.5 - 2.0 eq.) and equivalent amount of triethylamine (only used with amine salts) were added to a solution of epoxide (1.0 eq.) in anhydrous methanol (10.5-22 mL/mmol). The reaction mixture was allowed to stir at 60-65 °C for either 6 hours or overnight. It was concentrated *in vacuo* and the crude product was purified by flash column chromatography on silica gel to afford a mixture of Boc-protected hydroxyethylamine intermediate and cyclic byproduct.

HCl (4 M in dioxane, 2.3 – 4.1 eq.) was added to the isolated intermediate/byproduct in anhydrous DCM (7-19 mL/mmol). The reaction mixture was allowed to stir at room temperature overnight. The reaction was concentrated *in vacuo*, redissolved in MeOH and concentrated again. It was then suspended in EtOAc and allowed to stir at room temperature for 30 minutes. The precipitate was filtered and washed with EtOAc to afford the title compound.

General Procedure B: Boc-Protection

Amine 32a/32b (1.0 eq.) and triethylamine (1.5 eq.) were dissolved in anhydrous DCM (1.4 mL/mmol) under N_2 atmosphere. The mixture was cooled to 0 °C and a solution of Boc_2O (1.2 eq.) in anhydrous DCM (1.0 mL/mmol) was slowly added over 2-3 min. The reaction mixture was warmed to room temperature and allowed to stir for 4 hours. After this time, it was diluted with DCM, and washed with water and brine. The organic layer was dried over $MgSO_4$, filtered and concentrated *in vacuo*. The crude product was purified by flash column chromatography on silica gel (10% EtOAc in hexane) to afford the title compound.

General Procedure C: Sonogashira Coupling

$Pd(PPh_3)Cl_2$ (0.02 eq.), CuI (0.03 eq.) and ethynyltrimethylsilane (1.25 eq.) were added to the bromo intermediate (1.0 eq.) in triethylamine (1.8-1.9 mL/mmol) under a N_2 atmosphere. The reaction mixture was heated to 70 °C and allowed to stir overnight. The reaction mixture was diluted with EtOAc, filtered through celite and concentrated *in vacuo*. It was then dissolved in MeOH (12.0-12.6 mL/mmol), and K_2CO_3 (5.0 eq.) was added. The resultant reaction mixture was allowed to stir at room temperature for 3 hours under a N_2 atmosphere. After this time, the mixture was filtered through celite and concentrated *in vacuo.* The crude product was purified by flash column chromatography on silica gel.

General Procedure D: Boc-Deprotection

HCl (4 M in dioxane, 5.0 eq.) was added to a solution of Boc-protected amine (1.0 eq.) in anhydrous MeOH (42 mL/mmol). The reaction mixture was allowed to stir at room temperature overnight. It was concentrated *in vacuo*, redissolved in MeOH and concentrated again. It was then suspended in EtOAc and allowed to stir at room temperature for 30 minutes. The precipitate was filtered and washed with EtOAc to afford the title compound.

General Procedure E: Amide Coupling

A mixture of HATU (1.1-1.4 eq.) and amine/amine salt (1.0 eq.) in DMSO (16 - 80 mL/mmol) was allowed to stir for 15 min at room temperature. Acid (1.1-1.4 eq.) and 4-ethylmorpholine (3.5-3.9 eq.) were added and the reaction was allowed to stir at room temperature overnight in the absence of light. It was quenched with sat. aq. $NaHCO_3$ and extracted with EtOAc (3x). The combined organic extracts were washed with water and brine. They were dried over $MgSO_4$, filtered and concentrated *in vacuo*. The crude product was purified by flash column chromatography on silica gel (4-10% MeOH in DCM) to afford the title compound.

1.3 Synthesis

(2*R*,3*S*)-3-Amino-4-phenyl-1-((4-(3-(trifluoromethyl)-3H-diazirin-3-yl)benzyl)amino)butan-2-ol hydrochloride salt (5)

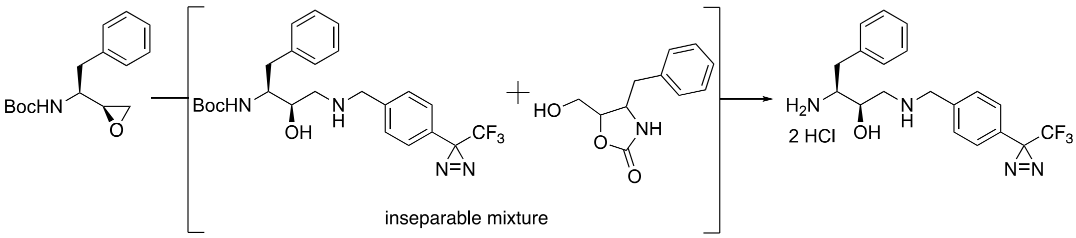

General procedure A was implemented with (4-(3-(trifluoromethyl)-3*H*-diazirin-3-yl)phenyl)methanamine hydrochloride salt (0.275 g, 1.09 mmol), triethylamine (0.15 mL, 1.09 mmol), *tert*-butyl ((*S*)-1-((*S*)-oxiran-2-yl)-2-phenylethyl)carbamate **2** (0.192 g, 0.73 mmol) and anhydrous methanol (15 mL). The reaction mixture was allowed to stir at 60 °C for 6 hours in the absence of light. It was concentrated *in vacuo* and the crude product was purified by flash column chromatography on silica gel (1-3% MeOH in DCM). A mixture of Boc-protected intermediate **3** and byproduct **4** was isolated as a white solid.

For Boc-deprotection, the crude mixture was dissolved in anhydrous DCM (5 mL) and treated with HCl (4 M in dioxane, 0.42 mL, 1.68 mmol). The title compound was obtained as a white solid (0.068 g, 21%). Following product precipitation and collection by filtration, the filtrate was concentrated *in vacuo* allowing isolation of clean cyclic product **5**.

Product **5** characterisation

^{1}H NMR (400 MHz, CD_3OD) δ 7.64 (d, *J* = 8.1 Hz, 2H), 7.39 – 7.25 (m, 7H), 4.38 – 4.21 (m, 3H), 3.74 – 3.65 (m, 1H), 3.23 (app. d, *J* = 11.9 Hz, 1H), 3.08 – 2.97 (m, 2H), 2.97 – 2.85 (m, 1H); ^{13}C NMR (101 MHz, CD_3OD) δ 136.5, 134.2, 132.1, 131.4, 130.8, 130.3, 128.7, 128.4, 123.5 (q, J = 273.6 Hz), 76.4, 67.2, 57.0, 51.5, 49.3, 34.4; HRMS (ES+) *m/z*: calculated for $C_{19}H_{22}F_3N_4O$ $[M+H]^+$: 379.1740; found: 379.1740 (Diff 0.06 ppm).

Cyclic byproduct **4** characterisation

^{1}H NMR (400 MHz, DMSO-d_6) δ 7.72 (s, 1H), 7.37 – 7.14 (m, 5H), 5.02 (t, *J* = 5.7 Hz, 1H), 4.15 (app. dd, *J* = 8.8, 4.8 Hz, 1H), 3.81 (app. q, *J* = 5.9 Hz, 1H), 3.40 – 3.34 (m, *J* = 5.5, 4.1 Hz, 1H), 3.26 – 3.14 (m, 1H), 2.82 (dd, *J* = 13.5, 5.5 Hz, 1H), 2.76 (dd, *J* = 13.5, 6.9 Hz, 1H); ^{13}C NMR (101 MHz, DMSO-d_6) δ 158.0, 136.5, 129.5, 128.4, 126.6, 80.3, 61.8, 53.9, 40.4; HRMS (ES+) *m/z*: calculated for $C_{11}H_{13}NO_3Na$ $[M+Na]^+$: 230.0788; found: 230.0784 (Diff 1.58 ppm).

(2*R*,3*S*)-3-Amino-4-phenyl-1-(((*R*)-1-phenylethyl)amino)butan-2-ol hydrochloride salt (19)

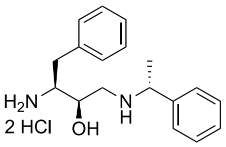

General procedure A was implemented with (*R*)-1-phenylethan-1-amine (0.97 mL, 7.6 mmol), *tert*-butyl ((*S*)-1-((*S*)-oxiran-2-yl)-2-phenylethyl)carbamate **2** (1.00 g, 3.8 mmol) and anhydrous methanol (40 mL). The reaction mixture was allowed to stir at 65 °C overnight. It was concentrated *in vacuo* and

the crude product was purified by flash column chromatography on silica gel (1% MeOH in EtOAc). A mixture of Boc-protected intermediate **18** and byproduct was isolated as a white solid.

For Boc-deprotection, the crude **18** was dissolved in anhydrous DCM (45 mL) and treated with HCl (4 M in dioxane, 3.11 mL, 12.43 mmol). The title compound (0.616 g, 45%) was obtained as a white solid. ^{1}H NMR (400 MHz, DMSO-d_6) δ 10.03 (br. s, 1H), 9.28 (br. s, 1H), 8.21 (br. s, 3H), 7.61 – 7.50 (m, 2H), 7.50 – 7.37 (m, 3H), 7.31 – 7.19 (m, 5H), 6.25 (d, *J* = 4.6 Hz, 1H), 4.39 – 4.27 (m, 1H), 4.26 – 4.16 (m, 1H), 3.50 (br. s, 1H), 2.92 (d, *J* = 12.4 Hz, 1H), 2.82 (d, *J* = 7.0 Hz, 2H), 2.49 – 2.44 (m, 1H), 1.60 (d, *J* = 6.8 Hz, 3H); ^{13}C NMR (101 MHz, CD_3OD) δ 137.1, 136.5, 130.8, 130.7, 130.24, 130.19, 128.8, 128.7, 67.3, 60.0, 57.1, 48.1, 34.5, 20.1; HRMS (ES+) *m/z*: calculated for $C_{18}H_{25}N_2O$ $[M+H]^+$: 285.1961; found: 285.1961 (Diff 0.14 ppm). Characterisation data consistent with the literature.[1]

(2*R*,3*S*)-3-Amino-1-(((*R*)-1-(4-ethynylphenyl)ethyl)amino)-4-phenylbutan-2-ol hydrochloride salt (27a)

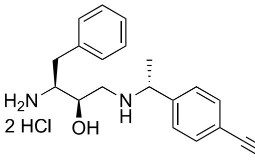

General procedure A was implemented with (*R*)-1-(4-ethynylphenyl)ethan-1-amine hydrochloride salt **25a** (0.250 g, 1.38 mmol), *tert*-butyl ((*S*)-1-((*S*)-oxiran-2-yl)-2-phenylethyl)carbamate **2** (0.181 g, 0.69 mmol), triethylamine (0.19 mL, 1.38 mmol) and anhydrous methanol (15 mL). The reaction mixture was allowed to stir at 65 °C overnight. It was concentrated *in vacuo* and the crude product was purified by flash column chromatography on silica gel (2% MeOH in EtOAc). A mixture of Boc-protected intermediate **26a** and byproduct was isolated as a white solid.

For Boc-deprotection, the crude **26a** was dissolved in anhydrous DCM (10 mL) and treated with HCl (4 M in dioxane, 0.47 mL, 1.90 mmol). The title compound (0.048 g, 18%) was obtained as a white solid. ^{1}H NMR (500 MHz, CD_3OD) δ 7.56 (d, *J* = 8.3 Hz, 2H), 7.45 (d, *J* = 8.3 Hz, 2H), 7.33 – 7.20 (m, 5H), 4.44 (q, *J* = 6.8 Hz, 1H), 4.25 (dt, *J* = 11.0, 2.6 Hz, 1H), 3.70 – 3.66 (m, 1H), 3.66 (s, 1H), 3.03 (dd, *J* = 12.7, 2.6 Hz, 1H), 2.96 (dd, *J* = 14.3, 7.7 Hz, 1H), 2.85 (dd, *J* = 14.3, 7.6 Hz, 1H), 2.62 (dd, *J* = 12.7, 11.0 Hz, 1H), 1.68 (d, *J* = 6.8 Hz, 3H); ^{13}C NMR (126 MHz, CD_3OD) δ 137.5, 136.5, 134.1, 130.2, 130.1, 129.0, 128.7, 125.2, 83.4, 80.3, 67.3, 59.6, 57.0, 48.1, 34.5, 19.9; HRMS (ES+) *m/z*: calculated for $C_{20}H_{25}N_2O$ $[M+H]^+$: 309.1961; found: 309.1960 (Diff 0.45 ppm).

(2*R*,3*S*)-3-Amino-1-(((*R*)-1-(3-ethynylphenyl)ethyl)amino)-4-phenylbutan-2-ol hydrochloride salt (27b)

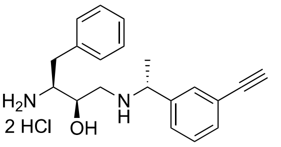

General procedure A was implemented with (*R*)-1-(3-ethynylphenyl)ethan-1-amine hydrochloride salt **25b** (0.600 g, 3.30 mmol), *tert*-butyl ((*S*)-1-((*S*)-oxiran-2-yl)-2-phenylethyl)carbamate **2** (0.435 g, 1.65 mmol), triethylamine (0.46 mL, 3.30 mmol) and anhydrous methanol (30 mL). The reaction mixture was allowed to stir at 65 °C overnight. It was concentrated *in vacuo* and the crude product was purified by flash column chromatography on silica gel (2% MeOH in EtOAc). A mixture of Boc-protected intermediate **26b** and byproduct was isolated as a white solid.

For Boc-deprotection, the crude **26b** was dissolved in anhydrous DCM (30 mL) and treated with HCl (4 M in dioxane, 1.19 mL, 4.77 mmol). The title compound (0.214 g, 34%) was obtained as a white solid. ^{1}H NMR (400 MHz, CD_3OD) δ 7.63 – 7.54 (m, 2H), 7.54 – 7.42 (m, 2H), 7.38 – 7.14 (m, 5H), 4.43 (q, *J* = 6.8 Hz, 1H), 4.27 (dt, *J* = 11.0, 2.4 Hz, 1H), 3.72 – 3.66 (m, 1H), 3.66 (s, 1H), 3.05 (dd, *J* = 12.6, 2.4 Hz, 1H), 2.97 (dd, *J* = 14.3, 7.6 Hz, 1H), 2.85 (dd, *J* = 14.3, 7.6 Hz, 1H), 2.62 (dd, *J* = 12.6, 11.0 Hz, 1H), 1.68 (d, *J* = 6.8 Hz, 3H); ^{13}C NMR (101 MHz, CD_3OD) δ 136.3, 135.1, 132.8, 130.9, 129.5, 128.80, 128.75, 127.7, 127.3, 123.8, 82.2, 78.8, 66.0, 58.2, 55.6, 46.9, 33.1, 18.7; HRMS (CI+) *m/z*: calculated for $C_{20}H_{25}N_2O$ $[M+H]^+$: 309.1961; found: 309.1959 (Diff 0.78 ppm).

(2*R*,3*S*)-3-Amino-4-(4-ethynylphenyl)-1-(((*R*)-1-phenylethyl)amino)butan-2-ol hydrochloride salt (36)

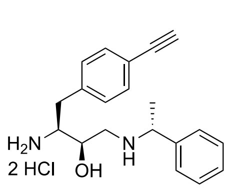

General procedure A was implemented with (*R*)-1-phenylethan-1-amine (0.14 mL, 1.06 mmol), *tert*-butyl ((*S*)-2-(4-ethynylphenyl)-1-((*S*)-oxiran-2-yl)ethyl)carbamate **34** (0.152 g, 0.53 mmol) and anhydrous methanol (10 mL). The reaction mixture was allowed to stir at 65 °C overnight. It was concentrated *in vacuo* and the crude product was purified by flash column chromatography on silica gel (2% MeOH in EtOAc). A mixture of Boc-protected intermediate **35** and byproduct was isolated as a white solid.

For Boc-deprotection, the crude **35** was dissolved in anhydrous DCM (10 mL) and treated with HCl (4 M in dioxane, 0.54 mL, 2.15 mmol). The title compound (0.089 g, 44%) was obtained as a white solid. ^{1}H NMR (400 MHz, CD_3OD) δ 7.51 – 7.43 (m, 5H), 7.39 (d, *J* = 8.1 Hz, 2H), 7.22 (d, *J* = 8.1 Hz, 2H), 4.41 (q, *J* = 6.8 Hz, 1H), 4.25 (dt, *J*= 10.9, 2.5 Hz, 1H), 3.67 (td, *J* = 7.5, 3.0 Hz, 1H), 3.55 (s, 1H), 3.02 (dd, *J* = 12.7, 2.5 Hz, 1H), 2.96 (dd, *J* = 14.4, 7.5 Hz, 1H), 2.84 (dd, *J* = 14.4, 7.6 Hz, 1H), 2.64 (dd, *J* = 12.7, 10.9 Hz, 1H), 1.69 (d, *J* = 6.8 Hz, 3H); ^{13}C NMR (101 MHz, CD_3OD) δ 137.3, 137.0, 133.8, 130.8, 130.7, 130.3, 128.7, 123.1, 84.0, 79.3, 67.3, 60.0, 56.7, 48.2, 34.3, 20.1; HRMS (ES+) *m/z*: calculated for $C_{20}H_{25}N_2O$ $[M+H]^+$: 309.1961; found: 309.1966 (Diff -1.49 ppm).

Methyl 4-(4-(prop-2-yn-1-yl)piperazine-1-carbonyl)benzoate (7)

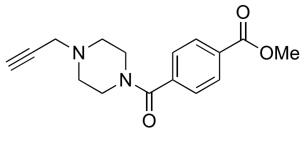

Oxalyl chloride (0.28 mL, 3.33 mmol, 2.0 eq.) was slowly added to a solution of 4-(methoxycarbonyl)benzoic acid **6** (0.300 g, 1.67 mmol, 1.0 eq.) in anhydrous DCM (2.5 mL). DMF (0.01 mL) was added, and the reaction mixture was allowed to stir at room temperature for 3 hours. The solvent was removed *in vacuo* to give a white solid which was dissolved in anhydrous THF (5 mL). The obtained solution was slowly added to a solution of 1-(prop-2-yn-1-yl)piperazine (0.248 g, 2.0 mmol, 1.5 eq.) in anhydrous THF (2.5 mL) cooled to 0 °C. The resulting reaction mixture was allowed to stir at room temperature for 1.5 hours. It was then concentrated *in vacuo*. The residue was diluted with water (40 mL) and extracted with EtOAc (3x50 mL). The organic layers were combined, washed with

brine (40 mL), dried over $MgSO_4$, filtered, and concentrated under reduced pressure to give the title compound (0.321 g, 67%) as a white solid. It was used in subsequent steps without further purification. ^{1}H NMR (400 MHz, $CDCl_3$) δ 8.09 (d, *J* = 8.0 Hz, 2H), 7.48 (d, *J* = 8.0 Hz, 2H), 3.94 (s, 3H), 3.85 (br. s, 2H), 3.43 (br. s, 2H), 3.37 (s, 2H), 2.67 (br. s, 2H), 2.52 (br. s, 2H), 2.30 (s, 1H); ^{13}C NMR (101 MHz, $CDCl_3$) δ 169.4, 166.5, 140.1, 131.3, 130.0, 127.2, 78.0, 74.1, 52.5, 52.0, 51.5, 47.5, 46.9, 42.0; HRMS (CI+) *m/z*: calculated for $C_{16}H_{19}N_2O_3$ $[M+H]^+$: 287.1390; found: 287.1382 (Diff 2.85 ppm).

4-(4-(Prop-2-yn-1-yl)piperazine-1-carbonyl)benzoic acid sodium chloride salt (8)

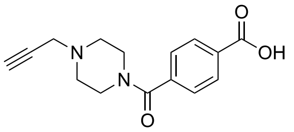

A solution of NaOH (0.58 mL, 2 M, 1.15 mmol, 1.2 eq.) was added to methyl 4-(4-(prop-2-yn-1-yl)piperazine-1-carbonyl)benzoate **7** (0.275 g, 0.96 mmol, 1.0 eq.) in MeOH (4 mL). It was heated to 50 °C and allowed to stir for 3 hours. It was then concentrated *in vacuo*. Water was added and the mixture was acidified to pH 2 using HCl (5 M). It was concentrated *in vacuo* to afford the title compound (containing 1.2 eq. NaCl) (0.293 g, 89%) as a white solid. It was used in subsequent steps without further purification.

^{1}H NMR (400 MHz, DMSO-d_6) δ 12.41 (br. s, 1H), 8.01 (d, *J* = 8.1 Hz, 2H), 7.57 (d, *J* = 8.1 Hz, 2H), 4.08 (s, 2H), 3.83 (s, 1H), 3.67 – 2.99 (m, 8H); ^{13}C NMR (101 MHz, DMSO-d_6) δ 168.4, 166.7, 138.8, 132.0, 129.5, 127.3, 81.3, 73.2, 49.90, 49.88, 44.3; HRMS (CI+) *m/z*: calculated for $C_{15}H_{17}N_2O_3$ $[M+H]^+$: 273.1234; found: 273.1228 (Diff 2.08 ppm).

Ethyl 3-oxohept-6-ynoate (10)

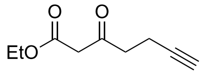

n-BuLi (42.81 mL, 2.5 M solution in hexanes, 107.0 mmol, 2.2 eq.) was added to diisopropylamine (15 mL, 107.0 mmol, 2.2 eq.) in anhydrous THF (100 mL) at -78 °C. The reaction mixture was allowed to stir for 15 minutes. Ethyl acetoacetate **9** (6.15 mL, 48.6 mmol, 1.0 eq.) was added dropwise. The mixture was warmed to 0 °C and allowed to stir for 30 minutes. Propargyl bromide (5.77 mL, 80% solution in toluene, 53.5 mmol, 1.1 eq.) was then added. The reaction mixture was warmed to room temperature and allowed to stir overnight. After this time, the reaction was poured into saturated aq. NH_4Cl and extracted with EtOAc (3x). The combined organic extracts were washed with water and brine. They were dried over $MgSO_4$, filtered, and concentrated *in vacuo* to yield a crude brown oil which was purified by flash column chromatography on silica gel (10% EtOAc in hexane) to afford the title compound (6.07 g, 74%) as a yellow oil.

^{1}H NMR (400 MHz, $CDCl_3$) δ 4.20 (q, *J* = 7.1 Hz, 2H), 3.47 (s, 2H), 2.82 (t, *J* = 7.3 Hz, 2H), 2.48 (td, *J* = 7.3, 2.4 Hz, 2H), 1.97 (s, 1H), 1.29 (t, *J* = 7.1 Hz, 3H); ^{13}C NMR (101 MHz, $CDCl_3$) δ 200.7, 167.0, 82.7, 69.1, 61.6, 49.4, 41.7, 14.2, 13.0; HRMS (CI+) *m/z*: calculated for $C_9H_{13}O_3$ $[M+H]^+$: 169.0859; found: 169.0850 (Diff -2.98 ppm). Characterisation data consistent with the literature.[2,3]

Ethyl 2-(2-(but-3-yn-1-yl)-1,3-dioxolan-2-yl)acetate (11)

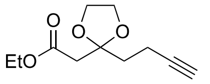

Triethyl orthoformate (11.79 mL, 70.87 mmol, 2.0 eq.) and ethylene glycol (7.90 mL, 141.74 mmol, 4.0 eq.) were added to ethyl 3-oxohept-6-ynoate **10** (5.96 g, 35.44 mmol, 1.0 eq.) under N_2 atmosphere. Tosic acid monohydrate (0.67 g, 3.54 mmol, 0.1 eq.) was added and the reaction mixture was allowed to stir at room temperature overnight. After this time, sat. aq. $NaHCO_3$ was added and the mixture was extracted with diethyl ether (3x). The combined organic extracts were washed with brine, dried over $MgSO_4$, filtered and concentrated *in vacuo*. The crude product was then purified by flash column chromatography on silica gel (20% EtOAc in hexane) to afford the title compound (6.72 g, 89%) as a yellow oil.

^{1}H NMR (400 MHz, $CDCl_3$) δ 4.16 (q, *J* = 7.1 Hz, 2H), 4.08 – 3.92 (m, 4H), 2.66 (s, 2H), 2.31 (t, *J* = 6.9 Hz, 2H), 2.17 – 2.05 (m, 2H), 1.93 (s, 1H), 1.27 (t, *J* = 7.1 Hz, 3H); ^{13}C NMR (101 MHz, $CDCl_3$) δ 169.3, 108.4, 84.1, 68.2, 65.4, 60.8, 42.9, 36.5, 14.3, 12.9; HRMS (CI+) *m/z*: calculated for $C_{11}H_{17}O_4$ $[M+H]^+$: 213.1121; found: 213.1127 (Diff 2.48 ppm). Characterisation data consistent with the literature.[2,3]

2-(2-(But-3-yn-1-yl)-1,3-dioxolan-2-yl)ethan-1-ol (12)

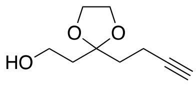

A solution of ethyl 2-(2-(but-3-yn-1-yl)-1,3-dioxolan-2-yl)acetate **11** (6.62 g, 31.19 mmol, 1.0 eq.) in anhydrous THF (22 mL) was added slowly to a stirred solution of $LiAlH_4$ (23.39 mL, 2 M in THF, 46.78 mmol, 1.5 eq.) in anhydrous THF (22 mL) at 0°C. The reaction mixture was heated to 80 °C and allowed to stir for 2 hours. It was then cooled to 0 °C and water (50 mL) was slowly added with vigorous stirring, followed by a 1 M KOH solution (50 mL). The biphasic solution was filtered over a small pad of silica which was subsequently washed with Et_2O. The organic phase of the filtrate was separated, washed with brine, dried over $MgSO_4$, filtered, and concentrated under reduced pressure to give the title compound (4.08 g, 77%) as a yellow oil.

^{1}H NMR (400 MHz, $CDCl_3$) δ 4.09 – 3.91 (m, 4H), 3.83 – 3.69 (m, 2H), 2.53 (br. s, 1H), 2.33 – 2.18 (m, 2H), 2.01 – 1.85 (m, 5H); ^{13}C NMR (101 MHz, $CDCl_3$) δ 111.2, 84.1, 68.4, 65.1, 58.8, 38.4, 36.0, 13.3; HRMS (CI+) *m/z*: calculated for $C_9H_{15}O_3$ $[M+H]^+$: 171.1016; found: 171.1009 (Diff -4.07 ppm). Characterisation data consistent with the literature.[3]

1-Hydroxyhept-6-yn-3-one (13)

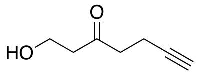

2-(2-(But-3-yn-1-yl)-1,3-dioxolan-2-yl)ethan-1-ol **12** (3.95 g, 23.21 mmol, 1.0 eq.) was dissolved in a mixture of acetone and water (5:1 v/v, 300 mL). Tosic acid monohydrate (1.104 g, 5.80 mmol, 0.25 eq.) was then added and the reaction mixture was allowed to stir at 75 °C for 6 hours. After this time, the reaction mixture was concentrated *in vacuo*. The residue was then dissolved in EtOAc, washed with saturated aq. $NaHCO_3$ solution, water and brine. The organic layer was dried over $MgSO_4$, filtered, and concentrated *in vacuo* to yield a crude oil. It was purified by flash column chromatography on silica gel (50% EtOAc in hexane) to afford the title compound (2.07 g, 71%) as a yellow oil.

^1H NMR (500 MHz, $CDCl_3$) δ 3.99 – 3.77 (m, 2H), 2.72 – 2.67 (m, 4H), 2.48 (td, *J* = 7.2, 2.7 Hz, 2H), 2.25 (br. s, 1H), 1.98 (t, *J* = 2.7 Hz, 1H); ^{13}C NMR (126 MHz, $CDCl_3$) δ 209.2, 82.9, 69.0, 57.8, 44.7, 41.9, 12.9; HRMS (CI+) *m/z*: calculated for $C_7H_{14}NO_3$ $[M+NH_4]^+$: 144.1019; found: 144.1021 (Diff 1.65 ppm). Characterisation data consistent with the literature.[3]

2-(3-(But-3-yn-1-yl)-3*H*-diazirin-3-yl)ethan-1-ol (14)

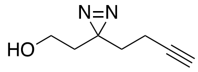

NH_3 (8.49 mL, 7 M in MeOH, 59.45 mmol, 15.0 eq.) was added to 1-hydroxyhept-6-yn-3-one **13** (0.500 g, 3.96 mmol, 1.0 eq.) at -10 °C in the absence of light under N_2 atmosphere. The reaction mixture was stirred at this temperature for 4.5 hours. A solution of hydroxylamine-*O*-sulfonic acid (0.583 g, 5.15 mmol, 1.3 eq.) in anhydrous methanol (3 mL) was added dropwise at -10 °C and the mixture was allowed to stir at this temperature for 1 hour. It was then warmed to room temperature and allowed to stir for 16 hours.

The formed slurry was filtered and washed with anhydrous methanol. The filtrate was then concentrated *in vacuo*. It was then redissolved in anhydrous methanol (3 mL) and triethylamine (4.09 mL, 29.33 mmol, 7.4 eq.) was added at 0 °C. A solution of I_2 (1.308 g, 5.15 mmol, 1.3 eq.) in anhydrous methanol (10 mL) was added dropwise at 0 °C. The reaction mixture was allowed to stir at 0 °C for 1 hour. It was then diluted with Et_2O (20 mL) and washed with brine (20 mL). The organic layer was dried over $MgSO_4$, filtered, and concentrated *in vacuo*. It was purified by flash column chromatography on silica gel (15-20% EtOAc in hexane) to afford the title compound (0.083 g, 15%) as a yellow oil.

^1H NMR (500 MHz, $CDCl_3$) δ 3.49 (t, *J* = 5.8 Hz, 2H), 2.04 (t, *J* = 6.2 Hz, 2H), 2.00 (s, 1H), 1.77 – 1.65 (m, 4H); ^{13}C NMR (126 MHz, $CDCl_3$) δ 83.0, 69.4, 57.5, 35.6, 32.8, 26.7, 13.4. Characterisation data consistent with the literature.[4]

3-(But-3-yn-1-yl)-3-(2-iodoethyl)-3*H*-diazirine (15)

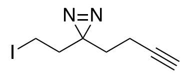

Iodine (0.324 g, 1.28 mmol, 1.2 eq.) was added to a solution of PPh_3 (0.307 g, 1.17 mmol, 1.1 eq.) and imidazole (0.217 g, 3.19 mmol, 3.0 eq.) in anhydrous DCM (6 mL) at 0 °C in the absence of light under N_2 atmosphere. It was allowed to stir for 15 minutes. A solution of 2-(3-(but-3-yn-1-yl)-3*H*-diazirin-3-yl)ethan-1-ol **14** (0.147 g, 1.06 mmol, 1.0 eq.) in anhydrous DCM (1.2 mL) was then added slowly. The reaction mixture was allowed to stir for 2 hours at 0 °C, followed by another 2 hours at room temperature. It was then quenched by addition of saturated aq. $Na_2S_2O_3$. The aqueous layer was extracted with DCM (3x). The combined organic extracts were washed with brine, dried over $MgSO_4$, filtered, and concentrated *in vacuo*. The crude was purified by flash column chromatography on silica gel (5% EtOAc in hexane) to afford the title compound (0.108 g, 41%) as a clear oil.

^1H NMR (400 MHz, $CDCl_3$) δ 2.89 (t, *J* = 7.4 Hz, 2H), 2.12 (t, *J* = 7.4 Hz, 2H), 2.06 – 1.93 (m, 3H), 1.68 (t, *J* = 7.0 Hz, 2H); ^{13}C NMR (101 MHz, $CDCl_3$) δ 82.6, 69.6, 37.7, 32.0, 28.8, 13.4, -3.9. Characterisation data consistent with the literature.[3]

Methyl 4-(2-(3-(but-3-yn-1-yl)-3*H*-diazirin-3-yl)ethoxy)benzoate (16)

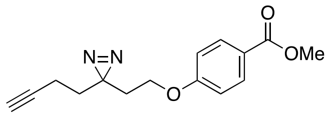

Methyl 4-hydroxybenzoate (0.059 g, 0.38 mmol, 1.0 eq.) was added to 3-(but-3-yn-1-yl)-3-(2-iodoethyl)-3*H*-diazirine **15** (0.105 g, 0.42 mmol, 1.1 eq.) in DMF (5 mL). K_2CO_3 (0.160 g, 1.15 mmol, 3.0 eq.) was then added and the reaction mixture was heated to 60 °C. It was allowed to stir at this temperature for 48 hours in the absence of light. Water was added and the aqueous layer was extracted with EtOAc. The organic layer was then washed with brine (3x), dried over $MgSO_4$, filtered, and concentrated *in vacuo*. The crude was purified by flash column chromatography on silica gel (5% EtOAc in hexane) to afford the title compound (0.024 g, 23%) as a yellow oil.

^{1}H NMR (400 MHz, $CDCl_3$) δ 8.01 (d, *J* = 8.4 Hz, 2H), 6.92 (d, *J* = 8.4 Hz, 2H), 3.93 – 3.83 (m, 5H), 2.09 (t, *J* = 7.3 Hz, 2H), 1.99 (s, 1H), 1.92 (t, *J* = 6.2 Hz, 2H), 1.76 (t, *J* = 7.3 Hz, 2H); ^{13}C NMR (101 MHz, $CDCl_3$) δ 166.9, 162.3, 131.8, 123.1, 114.2, 82.8, 69.4, 62.8, 52.0, 33.0, 32.8, 26.7, 13.4.

4-(2-(3-(But-3-yn-1-yl)-3*H*-diazirin-3-yl)ethoxy)benzoic acid (17)

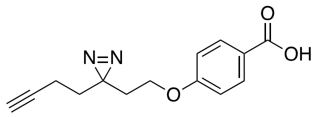

A solution of NaOH (0.10 mL, 2 M, 0.20 mmol, 1.5 eq.) was added to methyl 4-(2-(3-(but-3-yn-1-yl)-3*H*-diazirin-3-yl)ethoxy)benzoate **16** (0.036 g, 0.13 mmol, 1.0 eq.) in MeOH (1 mL). It was heated to 50 °C and allowed to stir for 3.5 hours. It was then cooled down to room temperature and left to stir overnight in the absence of light. More of the NaOH solution (0.3 mL, 2 M, 0.06 mmol, 0.5 eq.) was added. The reaction was heated to 50 °C and allowed to stir overnight. It was then concentrated *in vacuo*. Water was added and the mixture was acidified to pH 2 using HCl (5 M). The precipitate formed was filtered and washed with a small amount of water affording the title compound (0.007 g, 20%) as a white solid.

^{1}H NMR (500 MHz, CD_3OD) δ 7.97 (d, *J* = 8.9 Hz, 2H), 6.98 (d, *J* = 8.9 Hz, 2H), 3.93 (t, *J* = 6.1 Hz, 2H), 2.27 (t, *J* = 2.7 Hz, 1H), 2.08 (td, *J* = 7.4, 2.7 Hz, 2H), 1.91 (t, *J* = 6.1 Hz, 2H), 1.70 (t, *J* = 7.4 Hz, 2H); ^{13}C NMR (126 MHz, CD_3OD) δ 169.8, 163.9, 132.8, 124.4, 115.2, 83.7, 70.3, 64.1, 33.8, 33.7, 27.7, 13.8. Characterisation data consistent with the literature.[5]

***Tert*-Butyl (*R*)-(1-(4-bromophenyl)ethyl)carbamate (23a)**

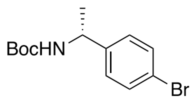

General procedure B was implemented with (*R*)-1-(4-bromophenyl)ethan-1-amine **22a** (1.5 mL, 10.42 mmol), triethylamine (2.18 mL, 15.63 mmol), DCM (15 mL) and Boc_2O (2.729 g, 12.50 mmol) in anhydrous DCM (10 mL). The title compound (2.210 g, 71%) was obtained as a white solid.

^{1}H NMR (500 MHz, CD_3OD) δ 7.45 (d, *J* = 8.4 Hz, 2H), 7.22 (d, *J* = 8.4 Hz, 2H), 4.68 - 4.43 (m, 1H), 1.42 (s, 9H), 1.37 (d, *J* = 7.1 Hz, 3H); ^{13}C NMR (126 MHz, CD_3OD) δ 157.6, 145.6, 132.4, 128.9, 121.4, 80.2, 51.0, 28.8, 22.9; HRMS (ES+) *m/z*: calculated for $C_{13}H_{18}{}^{79}BrNO_2Na$ $[M+Na]^+$: 322.0413; found: 322.0412 (Diff 0.35 ppm). Characterisation data consistent with the literature.[6]

Tert-Butyl (R)-(1-(3-bromophenyl)ethyl)carbamate (23)

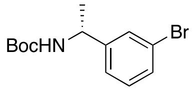

General procedure B was implemented with (*R*)-1-(3-bromophenyl)ethan-1-amine **22b** (1.5 mL, 10.50 mmol), triethylamine (2.20 mL, 15.75 mmol), DCM (15 mL) and Boc_2O (2.750 g, 12.60 mmol) in anhydrous DCM (10 mL). The title compound (2.819 g, 89%) was obtained as a white solid.
^1H NMR (400 MHz, CD_3OD) δ 7.47 (s, 1H), 7.37 (d, *J* = 7.6 Hz, 1H), 7.30 – 7.17 (m, 2H), 4.71 – 4.51 (m, 1H), 1.50 – 1.27 (m, 12H); ^{13}C NMR (101 MHz, CD_3OD) δ 157.6, 149.1, 131.2, 130.9, 130.0, 125.8, 123.4, 80.2, 51.1, 28.7, 23.0; HRMS (ES+) *m/z*: calculated for $C_{13}H_{18}{}^{79}BrNO_2Na$ $[M+Na]^+$: 322.0413; found: 322.0409 (Diff 1.28 ppm). Characterisation data consistent with the literature.[7]

Tert-Butyl (R)-(1-(4-ethynylphenyl)ethyl)carbamate (24a)

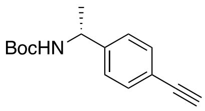

General procedure C was implemented with *tert*-butyl (*R*)-(1-(4-bromophenyl)ethyl)carbamate **23a** (1.000 g, 3.33 mmol), ethynyltrimethylsilane (0.58 mL, 4.16 mmol), $Pd(PPh_3)Cl_2$ (0.047 g, 0.07 mmol), CuI (0.019 g, 0.10 mmol) and triethylamine (6 mL), later followed by K_2CO_3 (2.302 g, 16.66 mmol) and MeOH (40 mL). The crude product was purified by flash column chromatography on silica gel (0-10% EtOAc in hexane) to afford the title compound (0.593 g, 73%) as a white solid.
^1H NMR (400 MHz, CD_3OD) δ 7.41 (d, *J* = 8.2 Hz, 2H), 7.28 (d, *J* = 8.2 Hz, 2H), 4.72 – 4.54 (m, 1H), 3.42 (s, 1H), 1.42 (s, 9H), 1.37 (d, *J* = 7.1 Hz, 3H); ^{13}C NMR (101 MHz, CD_3OD) δ 157.6, 147.1, 133.1, 127.0, 122.2, 84.3, 80.2, 78.3, 51.2, 28.8, 22.9; HRMS (ES+) *m/z*: calculated for $C_{15}H_{19}NO_2Na$ $[M+Na]^+$: 268.1308; found: 268.1305 (Diff 1.12 ppm).

Tert-Butyl (R)-(1-(3-ethynylphenyl)ethyl)carbamate (24b)

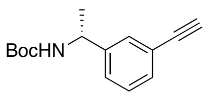

General procedure C was implemented with *tert*-butyl (*R*)-(1-(3-bromophenyl)ethyl)carbamate **23b** (1.900 g, 6.33 mmol), ethynyltrimethylsilane (1.10 mL, 7.91 mmol), $Pd(PPh_3)Cl_2$ (0.089 g, 0.13 mmol), CuI (0.036 g, 0.19 mmol) and triethylamine (12 mL), later followed by K_2CO_3 (4.370 g, 31.65 mmol) and MeOH (80 mL). The crude product was purified by flash column chromatography on silica gel (10% EtOAc in hexane) to afford the title compound (1.274 g, 82%) as a white solid.
^1H NMR (400 MHz, CD_3OD) δ 7.41 (s, 1H), 7.36 – 7.23 (m, 3H), 4.74 – 4.49 (m, 1H), 3.45 (s, 1H), 1.50 – 1.21 (m, 12H); ^{13}C NMR (101 MHz, CD_3OD) δ 157.6, 146.8, 131.4, 130.4, 129.5, 127.4, 123.8, 84.5, 80.2, 78.4, 51.1, 28.8, 23.0; HRMS (ES+) *m/z*: calculated for $C_{15}H_{19}NO_2Na$ $[M+Na]^+$: 268.1308; found: 268.1303 (Diff 1.86 ppm).

(*R*)-1-(4-Ethynylphenyl)ethan-1-amine hydrochloride salt (25a)

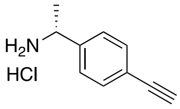

General procedure D was implemented with *tert*-butyl (*R*)-(1-(4-ethynylphenyl)ethyl)carbamate **24a** (0.588 g, 2.40 mmol), HCl (4 M in dioxane, 3.0 mL, 11.98 mmol) and anhydrous MeOH (100 mL). The title compound (0.365 g, 84%) was obtained as a white solid.

^{1}H NMR (500 MHz, CD_3OD) δ 7.60 – 7.52 (m, 2H), 7.51 – 7.42 (m, 2H), 4.49 (q, *J* = 6.9 Hz, 1H), 3.59 (s, 1H), 1.63 (d, *J* = 6.9 Hz, 3H); ^{13}C NMR (126 MHz, CD_3OD) δ 140.0, 133.8, 127.9, 124.7, 83.5, 79.9, 52.0, 20.5; HRMS (ES+) *m/z*: calculated for $C_{10}H_{12}N$ $[M+H]^+$: 146.0964; found: 146.0961 (Diff 2.23 ppm).

(*R*)-1-(3-Ethynylphenyl)ethan-1-amine hydrochloride salt (25b)

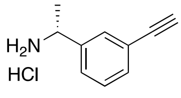

General procedure D was implemented with *tert*-butyl (*R*)-(1-(3-ethynylphenyl)ethyl)carbamate **24b** (1.173 g, 4.78 mmol), HCl (4 M in dioxane, 5.98 mL, 23.91 mmol) and anhydrous MeOH (200 mL). The title compound (0.657 g, 76%) was obtained as a white solid.

^{1}H NMR (400 MHz, CD_3OD) δ 7.60 (s, 1H), 7.55 – 7.36 (m, 3H), 4.47 (q, *J* = 6.9 Hz, 1H), 3.60 (s, 1H), 1.63 (d, *J* = 6.9 Hz, 3H); ^{13}C NMR (101 MHz, CD_3OD) δ 140.1, 133.6, 131.2, 130.5, 128.1, 124.9, 83.6, 79.8, 51.9, 20.6; HRMS (CI+) *m/z*: calculated for $C_{10}H_{12}N$ $[M+H]^+$: 146.0964; found: 146.0964 (Diff 0.18 ppm).

Methyl (*S*)-3-(4-bromophenyl)-2-((*tert*-butoxycarbonyl)amino)propanoate (29)

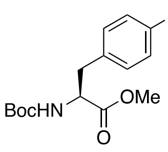

MeI (3.62 mL, 58.11 mmol, 5.0 eq.) was added to a solution of *N*-Boc-4-bromo-L-phenylalanine **28** (4.00 g, 11.62 mmol, 1.0 eq.) and $NaHCO_3$ (1.95 g, 23.24 mmol, 2.0 eq.) in anhydrous DMF (60 mL). The reaction mixture was allowed to stir at room temperature for 24 hours. Water (300 mL) was added and the mixture was extracted with EtOAc (3x). The combined organic extracts were washed with water (3x), brine (2x) and dried over $MgSO_4$. It was then filtered and concentrated *in vacuo* to give the title compound (3.73 g, 90%) as a yellow oil that solidifies left at room temperature overnight or after 4-5 hours when stored at 2 °C.

^{1}H NMR (400 MHz, $CDCl_3$) δ 7.41 (d, *J* = 8.2 Hz, 2H), 7.00 (d, *J* = 8.2 Hz, 2H), 5.00 (d, *J* = 7.2 Hz, 1H, NH), 4.62 – 4.49 (m, 1H), 3.71 (s, 3H), 3.09 (dd, *J* = 13.7, 5.6 Hz, 1H), 2.99 (dd, *J* = 13.7, 6.0 Hz, 1H), 1.42 (s, 9H); ^{13}C NMR (101 MHz, $CDCl_3$) δ 172.0, 155.0, 135.1, 131.6, 131.0, 121.0, 80.1, 54.2, 52.3, 37.8, 28.3; HRMS (ES+) *m/z*: calculated for $C_{15}H_{20}{}^{79}BrNO_4Na$ $[M+Na]^+$: 380.0468; found: 380.0469 (Diff -0.28 ppm). Characterisation data consistent with the literature.[8]

Tert-Butyl (_S_)-(1-(4-bromophenyl)-4-chloro-3-oxobutan-2-yl)carbamate (30)

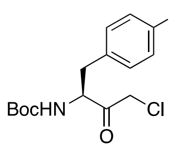

Methyl (*S*)-3-(4-bromophenyl)-2-((*tert*-butoxycarbonyl)amino)propanoate **29** (3.50 g, 9.77 mmol, 1.0 eq.), sodium chloroacetate (2.85 g, 24.43 mmol, 2.5 eq.) and triethylamine (2.72 mL, 19.54 mmol, 2.0 eq.) were dissolved in THF (22 mL). *Tert*-Butylmagnesium chloride (29.31 mL, 29.31 mmol, 3.0 eq.) was added to the solution at 0 °C under nitrogen atmosphere. It was allowed to stir overnight during which it warmed from 0 °C to room temperature. The reaction mixture was then added to a solution of citric acid (7.51 g, 39.08 mmol, 4.0 eq.) in water (22 mL) at 0 °C. The organic layer was separated and washed twice with an aqueous $NaHCO_3$/NaCl solution (18.21 g of $NaHCO_3$ and 65. 38 g NaCl in 400 mL of water) and once with brine. It was then dried over $MgSO_4$, filtered and concentrated *in vacuo* to afford the title compound (3.14 g, 85%) as a white solid.

^{1}H NMR (400 MHz, $CDCl_3$) δ 7.44 (d, *J* = 8.2 Hz, 2H), 7.05 (d, *J* = 8.2 Hz, 2H), 5.00 (d, *J* = 7.1 Hz, 1H, NH), 4.72 – 4.59 (m, 1H), 4.20 (d, *J* = 16.0 Hz, 1H), 4.08 (d, *J* = 16.0 Hz, 1H), 3.09 (dd, *J* = 14.0, 6.4 Hz, 1H), 2.93 (dd, *J* = 14.0, 7.3 Hz, 1H), 1.41 (s, 9H); ^{13}C NMR (101 MHz, $CDCl_3$) δ 201.1, 155.3, 134.9, 132.1, 131.0, 121.5, 80.9, 58.2, 47.3, 37.0, 28.4; HRMS (ES+) *m/z*: calculated for $C_{15}H_{19}{}^{79}BrClNO_3Na$ [M+Na]$^+$: 398.0129; found: 398.0128 (Diff 0.26 ppm).

Tert-Butyl ((2_S_,3_S_)-1-(4-bromophenyl)-4-chloro-3-hydroxybutan-2-yl)carbamate (31)

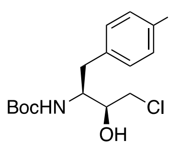

Sodium borohydride (0.771 g, 20.38 mmol, 2.5 eq.) was added in portions to a suspension of *tert*-butyl (*S*)-(1-(4-bromophenyl)-4-chloro-3-oxobutan-2-yl)carbamate **30** (3.07 g, 8.15 g, 1.0 eq.) in ethanol (63 mL) at -78 °C. The reaction mixture was allowed to stir at room temperature for 1 hour. Water was then added, and the mixture was acidified to pH 2 with a 2 M HCl solution at 0 °C. The precipitate was collected by filtration and washed with cold ethanol. It was recrystallised from hot EtOAc and collected by filtration. The title compound (0.971 g, 31%) was obtained as a white solid in 98:2 d.r. (HPLC).

^{1}H NMR (400 MHz, $CDCl_3$) δ 7.43 (d, *J* = 8.3 Hz, 2H), 7.10 (d, *J* = 8.3 Hz, 2H), 4.54 (d, *J* = 6.4 Hz, 1H, NH), 3.94 – 3.74 (m, 2H), 3.67 (dd, *J* = 11.1, 3.0 Hz, 1H), 3.58 (dd, *J* = 11.1, 7.3 Hz, 1H), 3.06 (br. s, 1H), 2.95 (dd, *J* = 14.1, 4.4 Hz, 1H), 2.92 – 2.82 (m, 1H), 1.37 (s, 9H); ^{13}C NMR (101 MHz, $CDCl_3$) δ 155.9, 136.6, 131.8, 131.4, 120.7, 80.3, 73.6, 54.3, 47.7, 35.4, 28.4; HRMS (ES+) *m/z*: calculated for $C_{15}H_{21}{}^{79}BrClNO_3Na$ [M+Na]$^+$: 400.0286; found: 400.0281 (Diff 1.14 ppm); 98:2 d.r. by HPLC R_t(major) = 10.290 min and R_t(minor) = 10.624 min.

Tert-Butyl ((S)-2-(4-bromophenyl)-1-((S)-oxiran-2-yl)ethyl)carbamate (32)

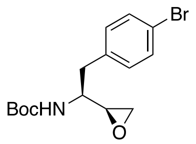

KOH (1.58 mL, 1 M in EtOH, 1.58 mmol, 1.5 eq.) was added to *tert*-butyl ((2*S*,3*S*)-1-(4-bromophenyl)-4-chloro-3-hydroxybutan-2-yl)carbamate **32** (0.400 g, 1.06 mmol, 2.0 eq.) in ethanol (16 mL). The reaction mixture was allowed to stir for 1 hour at room temperature. It was then concentrated *in vacuo*, diluted with water and extracted with DCM. The organic extract was dried over $MgSO_4$, filtered and concentrated *in vacuo* to afford the title compound (0.330 g, 91%) as white solid.

^{1}H NMR (400 MHz, $CDCl_3$) δ 7.43 (d, *J* = 8.3 Hz, 2H), 7.10 (d, *J* = 8.3 Hz, 2H), 4.44 (d, *J* = 7.8 Hz, 1H, NH), 3.72 – 3.55 (m, 1H), 2.96 – 2.87 (m, 2H), 2.86 – 2.64 (m, 3H), 1.38 (s, 9H); ^{13}C NMR (101 MHz, $CDCl_3$) δ 155.3, 136.0, 131.8, 131.3, 120.8, 80.0, 53.3, 52.7, 47.0, 37.2, 28.4; HRMS (ES+) *m/z*: calculated for $C_{15}H_{20}{}^{79}BrNO_3Na$ $[M+Na]^+$: 364.0519; found: 364.0519 (Diff -0.06 ppm).

Tert-Butyl ((S)-1-((S)-oxiran-2-yl)-2-(4-((trimethylsilyl)ethynyl)phenyl)ethyl) carbamate (33)

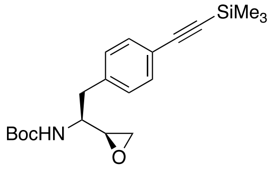

Ethynyltrimethylsilane (0.12 mL, 0.83 mmol, 1.25 eq.) was added to a stirred solution of *tert*-butyl ((*S*)-2-(4-bromophenyl)-1-((*S*)-oxiran-2-yl)ethyl)carbamate **32** (0.227 g, 0.66 mmol, 1.0 eq.), $Pd(PPh_3)Cl_2$ (0.023 g, 0.03 mmol, 0.05 eq.) and CuI (0.006 g, 0.03 mmol, 0.05 eq.) in triethylamine (5 mL) under N_2 atmosphere. The reaction mixture was heated to 70 °C and allowed to stir at this temperature overnight. The reaction mixture was diluted with water and extracted with Et_2O (2x). The organic extracts were washed with water (2x) and brine, dried over $MgSO_4$, filtered and concentrated *in vacuo*. The crude product was purified by flash column chromatography on silica gel (0-10% EtOAc in hexane) to afford the title compound (0.101 g, 43%) as an orange oil.

^{1}H NMR (400 MHz, $CDCl_3$) δ 7.41 (d, *J* = 8.1 Hz, 2H), 7.16 (d, *J* = 8.1 Hz, 2H), 4.39 (br. s, 1H), 3.67 (br. s, 1H), 2.96 (dd, *J* = 14.1, 5.1 Hz, 1H), 2.93 – 2.70 (m, 4H), 1.39 (s, 9H), 0.24 (s, 9H); ^{13}C NMR (101 MHz, $CDCl_3$) δ 155.3, 137.5, 132.3, 129.5, 121.7, 105.0, 94.4, 79.9, 53.3, 52.6, 47.1, 37.6, 28.4, 0.1; HRMS (ES+) *m/z*: calculated for $C_{20}H_{29}NO_3SiNa$ $[M+Na]^+$: 382.1809; found: 382.1807 (Diff 0.5 ppm).

Tert-Butyl ((S)-2-(4-ethynylphenyl)-1-((S)-oxiran-2-yl)ethyl)carbamate (34)

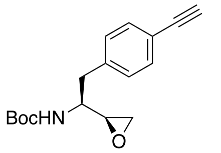

TBAF (0.63 mL, 0.62 mmol, 1.1 eq.) was added to *tert*-butyl ((*S*)-1-((*S*)-oxiran-2-yl)-2-(4-((trimethylsilyl)ethynyl)phenyl)ethyl) carbamate **33** (0.204 g, 0.57 mmol, 1.0 eq.) in THF (10 mL). The reaction mixture was allowed to stir at room temperature for 15 minutes. It was then concentrated *in vacuo* and purified by flash column chromatography on silica gel (0-10% EtOAc in hexane) to afford the title compound (0.126 g, 77%) as a white solid.

^1H NMR (400 MHz, $CDCl_3$) δ 7.44 (d, *J* = 8.1 Hz, 2H), 7.18 (d, *J* = 8.1 Hz, 2H), 4.42 (br. s, 1H), 3.69 (br. s, 1H), 3.06 (s, 1H), 2.97 (dd, *J* = 14.0, 5.1 Hz, 1H), 2.93 – 2.70 (m, 4H), 1.38 (s, 9H); ^{13}C NMR (101 MHz, $CDCl_3$) δ 155.3, 137.9, 132.4, 129.6, 120.6, 83.6, 80.0, 77.3, 53.3, 52.7, 47.0, 37.6, 28.4; HRMS (ES+) *m/z*: calculated for $C_{17}H_{21}NO_3Na$ [M+Na]$^+$: 310.1414; found: 310.1415 (Diff -0.44 ppm).

N-((2S,3R)-3-Hydroxy-1-phenyl-4-((4-(3-(trifluoromethyl)-3H-diazirin-3-yl)benzyl)amino)butan-2-yl)-4-(4-(prop-2-yn-1-yl)piperazine-1-carbonyl)benzamide (1a)

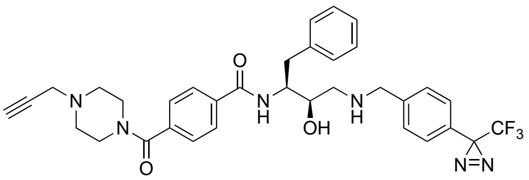

General procedure E was implemented with (2*R*,3*S*)-3-amino-4-phenyl-1-((4-(3-(trifluoromethyl)-3*H*-diazirin-3-yl)benzyl)amino)butan-2-ol hydrochloride salt **5** (0.065 g, 0.14 mmol), 4-(4-(prop-2-yn-1-yl)piperazine-1-carbonyl)benzoic acid sodium chloride salt **8** (0.064 g, 0.19 mmol), HATU (0.071 g, 0.19 mmol), 4-ethylmorpholine (0.07 mL, 0.54 mmol) and DMSO (5 mL). The title compound (0.031 g, 34%) was isolated as a white solid.

IR (solid) ν_{max}/cm^{-1} 3294 (m), 2917 (m), 2824 (m), 1700 (m), 1623 (s), 1536 (s), 1141 (s); ^1H NMR (400 MHz, CD_3OD) δ 7.64 (d, *J* = 8.1 Hz, 2H), 7.44 (m, 4H), 7.29 – 7.16 (m, 6H), 7.16 – 7.09 (m, 1H), 4.34 – 4.22 (m, 1H), 3.92 – 3.72 (m, 5H), 3.44 (br. s, 2H), 3.37 (d, *J* = 1.7 Hz, 2H), 3.22 (dd, *J* = 14.0, 4.0 Hz, 1H), 2.90 – 2.43 (m, 8H); ^{13}C NMR (101 MHz, CD_3OD) δ 171.4, 169.3, 143.1, 140.1, 139.5, 137.3, 130.33, 130.30, 129.3, 128.7, 128.6, 128.1, 127.7, 127.3, 123.6 (q, *J* = 273.8 Hz), 79.5, 78.7, 75.3, 73.1, 56.4, 53.8, 52.7, 52.3, 47.2, 43.0, 37.5; HRMS (ES+) *m/z*: calculated for $C_{34}H_{36}F_3N_6O_3$ [M+H]$^+$: 633.2796; found: 633.2803 (Diff -1.18 ppm); purity by HPLC 92% (R_t = 6.660 min).

4-(2-(3-(But-3-yn-1-yl)-3H-diazirin-3-yl)ethoxy)-N-((2S,3R)-3-hydroxy-1-phenyl-4-(((R)-1-phenylethyl)amino)butan-2-yl)benzamide (1b)

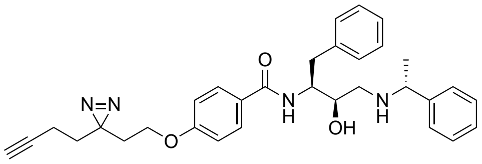

General procedure E was implemented with (2*R*,3*S*)-3-amino-4-phenyl-1-(((*R*)-1-phenylethyl)amino)butan-2-ol hydrochloride salt **19** (0.009 g, 0.025 mmol), 4-(2-(3-(but-3-yn-1-yl)-3*H*-diazirin-3-yl)ethoxy)benzoic acid **17** (0.007 g, 0.027 mmol), HATU (0.010 g, 0.027 mmol), 4-ethylmorpholine (0.012 mL, 0.095 mmol) and DMSO (2 mL). The title compound (0.013 g, 98%) was isolated as a white solid.

IR (solid) ν_{max}/cm^{-1} 3294 (br), 2919 (s), 2851 (s), 1630 (s), 1606 (s), 1543 (s), 1503 (s), 1448 (s), 1251 (s), 699 (s); ^1H NMR (500 MHz, CD_3OD) δ 7.57 (d, *J* = 8.9 Hz, 2H), 7.37 – 7.07 (m, 10H), 6.92 (d, *J* = 8.9 Hz, 2H), 4.25 – 4.11 (m, 1H), 3.95 – 3.83 (m, 3H), 3.77 (td, *J* = 7.4, 3.0 Hz, 1H), 3.14 (dd, *J* = 13.8, 4.4 Hz, 1H), 2.80 (dd, *J* = 13.8, 3.4 Hz, 1H), 2.72 (dd, *J* = 12.6, 3.0 Hz, 1H), 2.57 (dd, *J* = 12.6, 7.4 Hz, 1H), 2.27 (t, *J* = 2.7 Hz, 1H), 2.07 (td, *J* = 7.4, 2.7 Hz, 2H), 1.89 (t, *J* = 6.0 Hz, 2H), 1.69 (t, *J* = 7.4 Hz, 2H), 1.45 (d, *J* = 6.7 Hz, 3H); ^{13}C NMR (126 MHz, CD_3OD) δ 169.9, 162.7, 144.1, 140.0, 130.2, 130.1, 129.8, 129.3, 128.7, 128.0, 127.9, 127.3, 115.2, 83.7, 72.3, 70.3, 64.0, 59.4, 56.3, 51.0, 37.7, 33.8, 33.7, 27.7, 23.1, 13.8;

HRMS (ES+) *m/z*: calculated for $C_{32}H_{37}N_4O_3$ $[M+H]^+$: 525.286; found: 525.2862 (Diff -0.35 ppm); purity by HPLC 97% (R_t = 8.849 min).

4-Benzoyl-*N*-((2*S*,3*R*)-3-hydroxy-1-phenyl-4-(((*R*)-1-phenylethyl)amino)butan-2-yl)benzamide (20a)

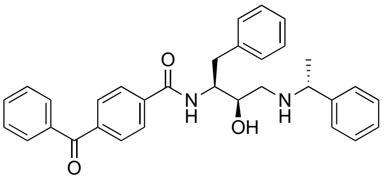

General procedure E was implemented with (2*R*,3*S*)-3-amino-4-phenyl-1-(((*R*)-1-phenylethyl)amino)butan-2-ol hydrochloride salt **19** (0.100 g, 0.28 mmol), 4-benzoylbenzoic acid **21** (0.085 g, 0.37 mmol), HATU (0.142 g, 0.37 mmol), 4-ethylmorpholine (0.14 mL, 1.09 mmol) and DMSO (5 mL). The title compound (0.086 g, 62%) was isolated as a white solid.

IR (solid) ν_{max}/cm^{-1} 3300 (br), 2925 (m), 2854 (m), 1638 (s), 1536 (s), 1277 (s), 697 (s); ^1H NMR (500 MHz, CD_3OD) δ 7.80 – 7.72 (m, 4H), 7.70 – 7.62 (m, 3H), 7.57 – 7.50 (m, 2H), 7.34 – 7.25 (m, 4H), 7.23 – 7.18 (m, 5H), 7.17 – 7.11 (m, 1H), 4.25 (ddd, *J* = 10.3, 7.5, 4.4 Hz, 1H), 3.86 – 3.74 (m, 2H), 3.16 (dd, *J* = 14.0, 4.4 Hz, 1H), 2.79 (dd, *J* = 14.0, 10.3 Hz, 1H), 2.73 (dd, *J* = 12.6, 3.0 Hz, 1H), 2.58 (dd, *J* = 12.6, 7.6 Hz, 1H), 1.41 (d, *J* = 6.7 Hz, 3H); ^{13}C NMR (126 MHz, CD_3OD) δ 197.6, 169.2, 145.1, 141.2, 140.0, 139.3, 138.4, 134.2, 131.0, 130.8, 130.3, 129.7, 129.6, 129.3, 128.4, 128.3, 127.9, 127.3, 72.4, 59.2, 56.6, 51.1, 37.7, 23.6; HRMS (ES+) *m/z*: calculated for $C_{32}H_{33}N_2O_3$ $[M+H]^+$: 493.2486; found: 493.2486 (Diff -0.06 ppm); purity by HPLC 98% (R_t = 7.964 min).

4-Benzoyl-*N*-((2*S*,3*R*)-4-(((*R*)-1-(4-ethynylphenyl)ethyl)amino)-3-hydroxy-1-phenylbutan-2-yl)benzamide (20b)

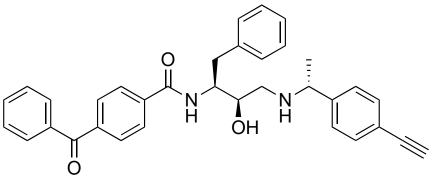

General procedure E was implemented with (2*R*,3*S*)-3-amino-1-(((*R*)-1-(4-ethynylphenyl)ethyl)amino)-4-phenylbutan-2-ol hydrochloride salt **27a** (0.041 g, 0.12 mmol), 4-benzoylbenzoic acid **21** (0.038 g, 0.11 mmol), HATU (0.054 g, 0.14 mmol), 4-ethylmorpholine (0.052 mL, 0.41 mmol) and DMSO (5 mL). The title compound (0.040 g, 72%) was isolated as a white solid.

IR (solid) ν_{max}/cm^{-1} 3292 (s), 2961 (m), 2831 (m), 1664 (s), 1633 (s), 1536 (s), 1279 (s), 697 (s); ^1H NMR (500 MHz, CD_3OD) δ 7.82 – 7.73 (m, 4H), 7.68 – 7.59 (m, 3H), 7.56 – 7.51 (m, 2H), 7.35 (d, *J* = 8.3 Hz, 2H), 7.30 (d, *J* = 8.3 Hz, 2H), 7.24 – 7.18 (m, 4H), 7.17 – 7.10 (m, 1H), 4.24 (ddd, *J* = 10.3, 7.8, 4.2 Hz, 1H), 3.87 (q, *J* = 6.6 Hz, 1H), 3.80 (td, *J* = 7.6, 2.9 Hz, 1H), 3.38 (s, 1H), 3.21 (dd, *J* = 14.0, 4.2 Hz, 1H), 2.79 (dd, *J* = 14.0, 10.3 Hz, 1H), 2.70 (dd, *J* = 12.7, 2.9 Hz, 1H), 2.55 (dd, *J* = 12.7, 7.6 Hz, 1H), 1.41 (d, *J* = 6.6 Hz, 3H); ^{13}C NMR (126 MHz, CD_3OD) δ 197.7, 169.3, 145.9, 141.2, 140.0, 139.3, 138.4, 134.1, 133.3, 131.1, 130.9, 130.3, 129.6, 129.3, 128.3, 128.1, 127.3, 122.8, 84.2, 78.6, 72.4, 58.8, 56.4, 51.0, 37.6, 23.4; HRMS (ES+) *m/z*: calculated for $C_{34}H_{33}N_2O_3$ $[M+H]^+$: 517.2486; found: 517.2480 (Diff 1.1 ppm); purity by HPLC 98% (R_t = 8.135 min).

4-Benzoyl-*N*-((2*S*,3*R*)-4-(((*R*)-1-(3-ethynylphenyl)ethyl)amino)-3-hydroxy-1-phenylbutan-2-yl)benzamide (20c)

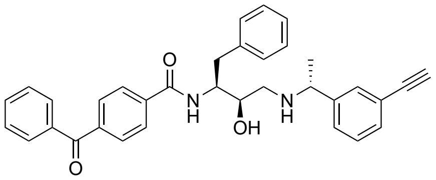

General procedure E was implemented with (2*R*,3*S*)-3-amino-1-(((*R*)-1-(3-ethynylphenyl)ethyl)amino)-4-phenylbutan-2-ol hydrochloride salt **27b** (0.100 g, 0.26 mmol), 4-benzoylbenzoic acid **21** (0.079 g, 0.35 mmol), HATU (0.132 g, 0.35 mmol), 4-ethylmorpholine (0.13 mL, 1.01 mmol) and DMSO (5 mL). The title compound (0.105 g, 78%) was isolated as a white solid.

IR (solid) ν_{max}/cm^{-1} 3287 (m), 2924 (m), 2854 (m), 1636 (s), 1599 (s), 1534 (s), 1277 (s), 697 (s); ^{1}H NMR (500 MHz, CD_3OD) δ 7.82 – 7.73 (m, 4H), 7.70 – 7.64 (m, 3H), 7.57 – 7.51 (m, 2H), 7.45 (s, 1H), 7.36 – 7.30 (m, 2H), 7.29 – 7.25 (m, 1H), 7.24 – 7.20 (m, 4H), 7.18 – 7.10 (m, 1H), 4.25 (ddd, *J* = 10.3, 7.6, 4.4 Hz, 1H), 3.86 (q, *J* = 6.6 Hz, 1H), 3.79 (td, *J* = 7.5, 3.0 Hz, 1H), 3.46 (s, 1H), 3.19 (dd, *J* = 14.0, 4.4 Hz, 1H), 2.80 – 2.75 (m, 1H), 2.72 (dd, *J* = 12.7, 3.0 Hz, 1H), 2.57 (dd, *J* = 12.7, 7.5 Hz, 1H), 1.41 (d, *J* = 6.6 Hz, 3H); ^{13}C NMR (126 MHz, CD_3OD) δ 197.7, 169.4, 145.5, 141.2, 140.0, 139.3, 138.4, 134.2, 132.1, 131.5, 131.1, 130.9, 130.3, 129.9, 129.6, 129.3, 128.5, 128.3, 127.3, 124.1, 84.4, 78.9, 72.3, 58.9, 56.5, 51.0, 37.7, 23.5; HRMS (ES+) *m/z*: calculated for $C_{34}H_{33}N_2O_3$ $[M+H]^+$: 517.2486; found: 517.2485 (Diff 0.13 ppm); purity by HPLC 100% (R_t = 8.220 min).

4-Benzoyl-*N*-((2*S*,3*R*)-1-(4-ethynylphenyl)-3-hydroxy-4-(((*R*)-1-phenylethyl)amino)butan-2-yl)benzamide (20d)

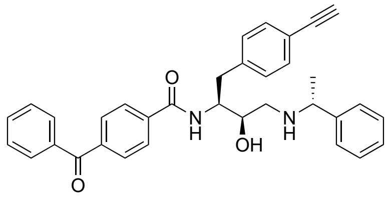

General procedure E was implemented with (2*R*,3*S*)-3-amino-4-(4-ethynylphenyl)-1-(((*R*)-1-phenylethyl)amino)butan-2-ol hydrochloride salt **36** (0.080 g, 0.23 mmol), 4-benzoylbenzoic acid **21** (0.063 g, 0.28 mmol), HATU (0.106 g, 0.28 mmol), 4-ethylmorpholine (0.10 mL, 0.81 mmol) and DMSO (5 mL). The title compound (0.091 g, 84%) was isolated as a cream solid.

IR (solid) ν_{max}/cm^{-1} 3285 (s), 2925 (m), 2854 (m), 1640 (s), 1598 (s), 1534 (s), 1276 (s), 698 (s); ^{1}H NMR (500 MHz, CD_3OD) δ 7.81 – 7.73 (m, 4H), 7.70 – 7.63 (m, 3H), 7.57 – 7.50 (m, 2H), 7.38 – 7.25 (m, 6H), 7.24 – 7.17 (m, 3H), 4.24 (ddd, *J* = 10.4, 7.7, 4.2 Hz, 1H), 3.89 (q, *J* = 6.6 Hz, 1H), 3.81 (td, *J* = 7.7, 3.0 Hz, 1H), 3.39 (s, 1H), 3.20 (dd, *J* = 14.0, 4.2 Hz, 1H), 2.81 – 2.77 (m, 1H), 2.74 (dd, *J* = 12.6, 3.0 Hz, 1H), 2.58 (dd, *J* = 12.6, 7.7 Hz, 1H), 1.44 (d, *J* = 6.6 Hz, 3H); ^{13}C NMR (126 MHz, CD_3OD) δ 197.7, 169.3, 144.3, 141.3, 141.0, 139.1, 138.4, 134.2, 133.0, 131.1, 130.9, 130.4, 129.8, 129.6, 128.6, 128.3, 128.0, 121.7, 84.3, 78.3, 72.2, 59.3, 56.3, 51.0, 37.5, 23.3; HRMS (ES+) *m/z*: calculated for $C_{34}H_{33}N_2O_3$ $[M+H]^+$: 517.2486; found: 517.2493 (Diff -1.41 ppm); purity by HPLC 94% (R_t = 8.130 min).

1.4 HPLC Data

Intermediate 31 showing its d.r.

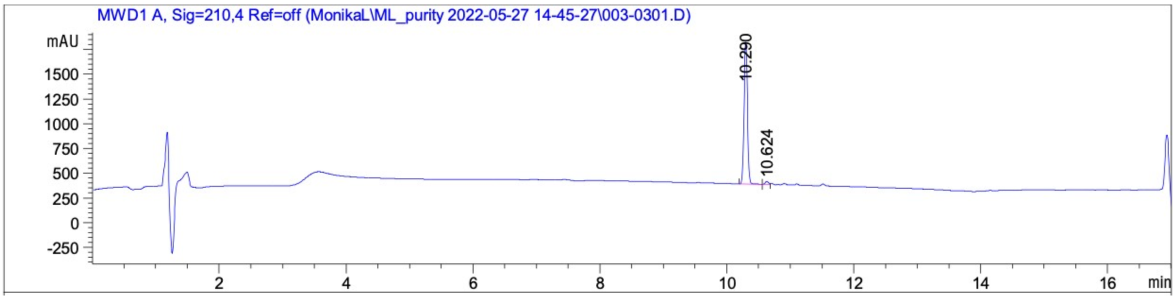

Signal 1: MWD1 A, Sig=210,4 Ref=off

Peak #	RetTime [min]	Type	Width [min]	Area [mAU*s]	Height [mAU]	Area %
1	10.290	BB	0.0588	5245.27100	1431.35974	98.0544
2	10.624	BV	0.0550	104.07484	29.59984	1.9456
Totals :				5349.34584	1460.95958	

Photoaffinity probe 1a

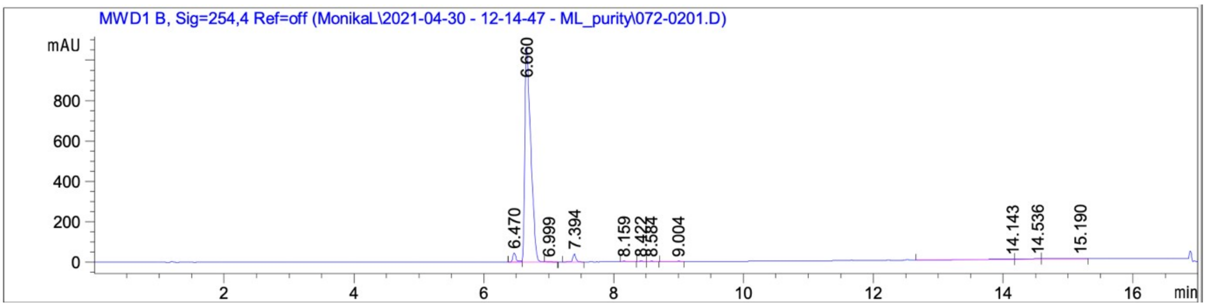

Signal 1: MWD1 B, Sig=254,4 Ref=off

Peak #	RetTime [min]	Type	Width [min]	Area [mAU*s]	Height [mAU]	Area %
1	6.470	BV E	0.0528	155.52432	44.43060	2.1191
2	6.660	VV R	0.0920	6748.99707	1063.70654	91.9605
3	6.999	VB E	0.0626	9.14498	2.19461	0.1246
4	7.394	VB R	0.0531	132.02696	39.42058	1.7990
5	8.159	BB	0.0589	12.52327	3.11762	0.1706
6	8.422	BV	0.0542	11.93946	3.46301	0.1627
7	8.584	VB	0.0635	9.87226	2.32488	0.1345
8	9.004	VB R	0.0580	8.02627	2.03553	0.1094
9	14.143	BV	0.4369	107.54355	2.96701	1.4654
10	14.536	VV	0.3098	65.01380	2.58145	0.8859
11	15.190	VV	0.5562	78.40503	1.70589	1.0683
Totals :				7339.01698	1167.94772	

Photoaffinity probe 1b

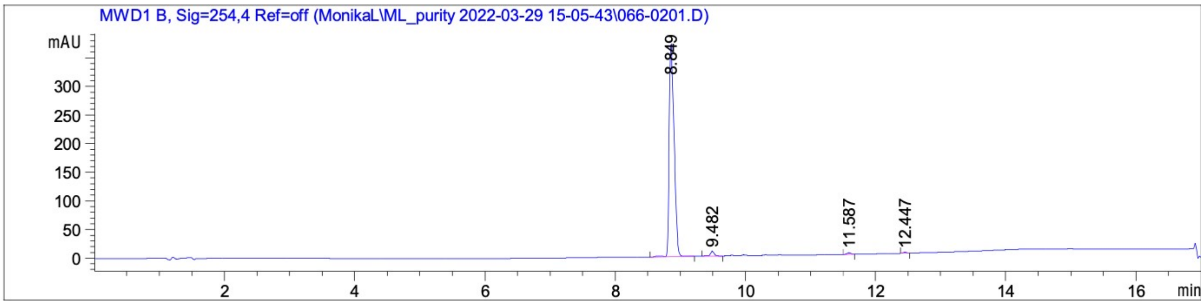

Signal 2: MWD1 B, Sig=254,4 Ref=off

Peak #	RetTime [min]	Type	Width [min]	Area [mAU*s]	Height [mAU]	Area %
1	8.849	VV R	0.0814	1959.37805	372.16815	97.4395
2	9.482	VB R	0.0638	35.76072	8.38044	1.7784
3	11.587	BB	0.0560	9.94796	2.76395	0.4947
4	12.447	BB	0.0533	5.77888	1.71711	0.2874

Totals : 2010.86562 385.02965

Photoaffinity probe 20a

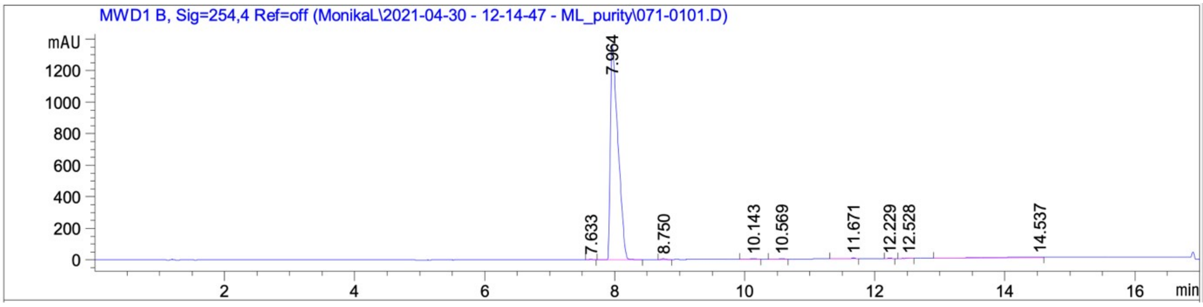

Signal 1: MWD1 B, Sig=254,4 Ref=off

Peak #	RetTime [min]	Type	Width [min]	Area [mAU*s]	Height [mAU]	Area %
1	7.633	BB	0.0525	9.91278	3.00181	0.0878
2	7.964	BV R	0.1166	1.10591e4	1363.75012	97.9203
3	8.750	BV	0.0548	15.99129	4.57191	0.1416
4	10.143	BV	0.1361	20.16890	2.26023	0.1786
5	10.569	VB	0.0692	12.30898	2.70079	0.1090
6	11.671	VB R	0.0607	21.00672	5.24509	0.1860
7	12.229	BB	0.0559	9.37069	2.60776	0.0830
8	12.528	VB R	0.0584	10.91750	3.00332	0.0967
9	14.537	BV	0.7622	135.20667	2.11406	1.1972

Totals : 1.12940e4 1389.25508

Photoaffinity probe 20b

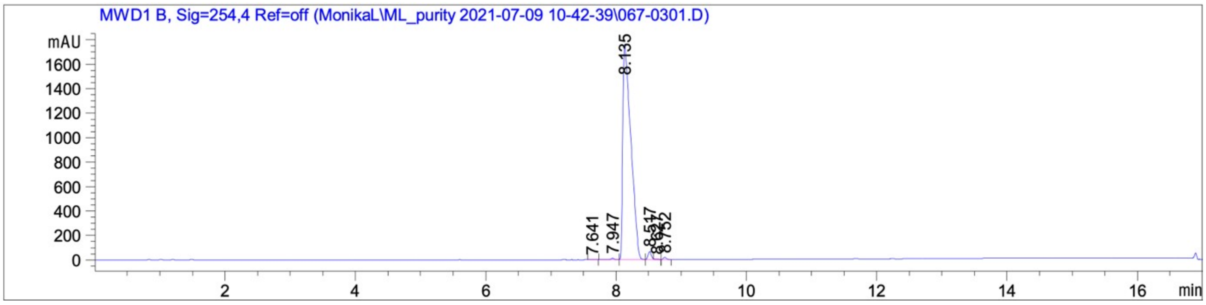

Signal 1: MWD1 B, Sig=254,4 Ref=off

Peak #	RetTime [min]	Type	Width [min]	Area [mAU*s]	Height [mAU]	Area %
1	7.641	BB	0.0530	6.66550	1.99384	0.0433
2	7.947	BB	0.0538	45.35667	12.66277	0.2948
3	8.135	BB	0.1234	1.50495e4	1765.99927	97.8176
4	8.517	BV R	0.0532	216.71225	64.49121	1.4086
5	8.627	VB E	0.0516	9.21928	2.86079	0.0599
6	8.752	BB	0.0516	57.81670	17.91956	0.3758

Totals : 1.53853e4 1865.92744

Photoaffinity probe 20c

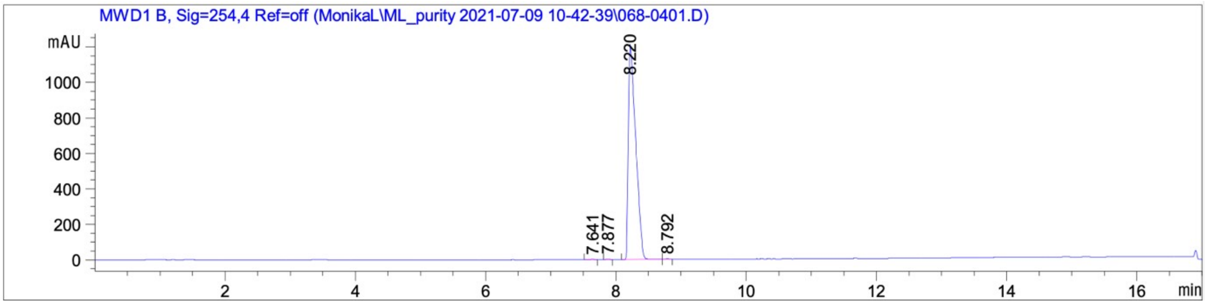

Signal 1: MWD1 B, Sig=254,4 Ref=off

Peak #	RetTime [min]	Type	Width [min]	Area [mAU*s]	Height [mAU]	Area %
1	7.641	BB	0.0516	10.12733	3.13819	0.1052
2	7.877	BV	0.0529	14.83896	4.44537	0.1541
3	8.220	BV R	0.1143	9591.29395	1212.43909	99.5919
4	8.792	BB	0.0537	14.33722	4.21418	0.1489

Totals : 9630.59746 1224.23682

Photoaffinity probe 20d

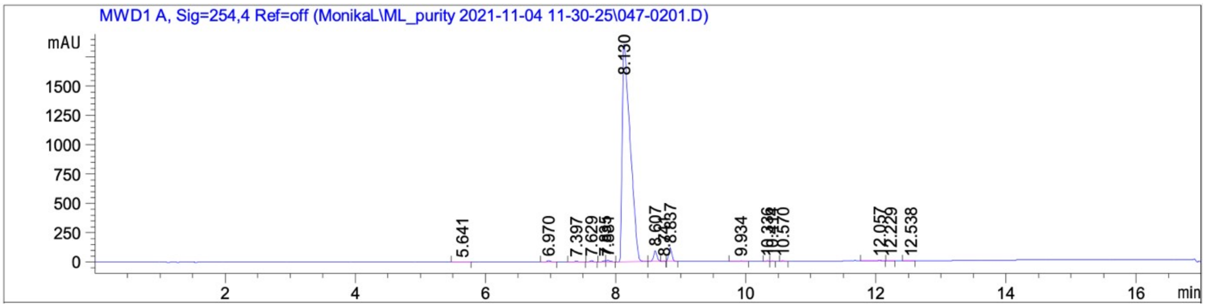

Signal 1: MWD1 A, Sig=254,4 Ref=off

Peak #	RetTime [min]	Type	Width [min]	Area [mAU*s]	Height [mAU]	Area %
1	5.641	VB R	0.0545	9.45215	2.71961	0.0566
2	6.970	BB	0.0529	37.41688	10.67830	0.2240
3	7.397	BB	0.0504	20.50224	6.22797	0.1228
4	7.629	BB	0.0527	25.14772	7.57601	0.1506
5	7.835	BV	0.0442	23.87157	8.72389	0.1429
6	7.881	VB	0.0603	42.36396	10.67136	0.2537
7	8.130	BB	0.1227	1.57318e4	1859.84705	94.1933
8	8.607	BV R	0.0572	334.46155	90.25653	2.0026
9	8.741	VV E	0.0477	11.03492	3.61040	0.0661
10	8.837	VB	0.0539	399.12918	116.64598	2.3898
11	9.934	VB R	0.0546	11.04241	3.17352	0.0661
12	10.336	BV	0.0548	7.20345	2.05995	0.0431
13	10.414	VV	0.0540	6.87288	1.91035	0.0412
14	10.570	VB	0.0586	7.26053	1.90088	0.0435
15	12.057	VB R	0.0620	20.78378	5.26997	0.1244
16	12.229	BB	0.0544	5.94780	1.71719	0.0356
17	12.538	VB	0.0636	7.31473	1.72179	0.0438

Totals : 1.67016e4 2134.71075

2 Biological Details

2.1. Antimalarial Activity Assay

P. falciparum cultures were prepared according to Trager and Jensen with slight modifications.[9] For the chemosensitivity assays, compounds were dissolved in DMSO and serial dilutions made with complete medium constituted by RPMI 1640 (EuroClone, Celbio) with the addition of 1% AlbuMax (Invitrogen, Milan, Italy), 0.01% hypoxanthine, 20 mM Hepes, and 2 mM glutamine. Asynchronous cultures with parasitaemia of 1-1.5 % and final hematocrit of 1 % were added and the plates were incubated for 72 h at 37 °C. Parasite growth was determined spectrophotometrically by measuring pLDH activity according to Makler with modifications.[10,11] 50 % inhibitory (IC_{50}) values are expressed as mean ± standard deviation (SD) of three different experiments, each performed in duplicate.

2.2. *Pf*PMX Peptide Cleavage Assay

Protease cleavage assays were performed with purified recombinant PfPMX produced from HEK cells following a published protocol with minor modifications.[12] Assays were performed in 30 µl volume in 384-well clear-bottom black plates with 50 nM of *Pf*PMX in final assay buffer (50 mM Acetate pH 5.5, 50 mM NaCl, 0.005 % TWEEN 20). Inhibitors were present at 1 µM and reactions contained a final 1 % DMSO concentration. Following 10 min preincubation of enzyme with inhibitors at 22 °C, 10 µM (final concentration) of synthetic peptide substrate (PfSUB1: DABCYL-G-SMLEVENDAE-G-EDANS) was added and fluorescence was measured using a SpectraMax Paradigm plate reader (Molecular Devices) set at 360 nm excitation and 490 nm emission wavelengths. Changes in Relative Fluorescence Unit (RFU) were measured every 2 min over 3 h by subtracting RFU from blank (no enzyme) for each timepoint. Assays were performed in triplicate and results were plotted using GraphPad Prism 10. Percentage of inhibition were calculated by plotting RFU for each condition after 180 min and normalized to the no inhibitor condition. P-values were calculated by unpaired student t-test.

3 Computational Details

3.1 Molecular Docking

To generate initial poses for molecular dynamics simulation we docked **49c** into an available crystal structure of plasmepsin X in complex with the inhibitor WM382 (PDB: 7TBC).[13] Docking was carried out in GOLD with a docking protocol validated by re-docking of the extracted WM382 ligand (**Supplementary Figure 9**).[14] We used default GOLD settings and the CHEMPLP scoring function to generate poses and rescored the generated poses using the GoldScore function. This afforded docking poses with RMSD values of < 1 Å with respect to the extracted ligand – a mean value of 0.583 Å was obtained for the top 10 generated poses indicating good performance of the docking protocol. To ensure likely interactions were captured in the initial pose, we constrained two hydrogen bonds between the hydroxylamine of **49c** and the aspartate catalytic dyad. In alignment with computational studies on similar BACE-1 inhibitors, we modelled the catalytic dyad as being double deprotonated and the ligand protonated at the hydroxylamine nitrogen.[15] The highest scoring pose was used as the initial structure for MD simulation.

To dock the probes described in this work, we used the representative structure of the final cluster of the lowest energy, determined by MM/PBSA methods, 100ns MD run of **49c**. Docking was achieved

using the same protocol as **49c**, however no constraints were used to generate docking poses. PLIP was used to detect non-covalent interactions between the probes and the binding site.

3.2 Molecular Dynamics Simulations

All molecular dynamics simulations were carried out using settings as published previously.[16] In short, the protein chain was parameterised using the pdb2gmx module in GROMACS version 5.1.4 using the Amber99-sb force-field.[17,18] Ligands were parameterised using Acpype as implemented in AmberTools22 using the GAFF force-field and AM1-BCC charges.[18] After concatenation of ligand and protein topologies, the complexes were solvated with TIP3P water molecules in a box with distance 2.0 nm from the protein volume and neutralised by addition of Na^+ ions. The system was energy minimised over 50000 steps using steepest descent minimisation. The complex was warmed to 300 K by NVT and NPT ensembles of 100ps each with positional restraints on the protein and ligand heavy atoms. Temperature and pressure were set to 300K and 1 bar controlled by the V-rescale modified Berendsen thermostat and the Parrinello-Rahman barostat, respectively.[19,20] We used the LINCS algorithm to constrain bonds to hydrogen, the Verlet cutoff-scheme for non-bonded interactions and the Particle Mesh Ewald (PME) scheme for electrostatic interactions. PME settings for FFT grid spacing was 0.16 nm and the interpolation order was 4. For each ligand, at 3 replicate production MD runs of 100ns were carried out using a timestep of 2 fs saving frames at every 10 ps. All MD simulations were undertaken on Nvidia A100 GPU nodes on Barkla, part of the High Performance Computing facilities at the University of Liverpool.

Trajectories were clustered using the MDtraj package to calculate the distance matrix between frames, and Scipy using agglomerative clustering with a distance cut-off of 2 Å.[21,22] Central structures of each cluster were analysed for binding interactions using PLIP.[23] Binding interactions identified in the top-5 most populated clusters were measured over the course of the simulation to identify stable binding interactions. For analysis of hydrogen-bonding interactions we used the Baker-Hubbard definition of donor-acceptor distance of < 2.5 Å and Donor-H-acceptor angle of > 120°.[24] Otherwise, we used the same interaction definitions as default in PLIP.

Binding free energy was calculated using the Molecular Mechanics/ Poisson-Boltzman Surface Area (MM/PBSA) approach.[25] Simply, ligand binding energy ($\Delta G_{binding}$) can be denoted as the difference in free energy between the a protein-ligand complex ($\Delta G_{Complex}$), and the sum of its components; protein and ligand free energy ($\Delta G_{protein}$, ΔG_{ligand}, respectively).

$$\Delta G_{binding} = \Delta G_{complex} - \Delta G_{protein} - \Delta G_{Ligand}$$

The MM/PBSA approach approximates binding free energy as the change in gas-phase interaction energy ($\Delta E_{MM,}$ sum of bonded, Van der Waal's (VdW) and electrostatic contributions) and the free energy change of solvation upon ligand binding (ΔG_{solv}), which in turn can be approximated from polar and apolar contributions to solvation free energy (ΔG_{polar} and ΔG_{apolar} respectively). In line with similar studies we neglect the entropic correction to $\Delta G_{binding}$ as it has been shown to have limited effect on the accuracy of free energy calculations.[26]

$$\Delta G_{binding} = \Delta G_{MM} - \Delta G_{solv}$$

$$\Delta G_{solv} = \Delta G_{polar} + \Delta G_{apolar}$$

MM/PBSA binding energy was calculated using the g_MMPBSA package.[27] Calculations were carried out for every 50 frames of the final 20 ns of the trajectory. We used the Poisson-Boltzman equation to calculate the polar component of solvation free energy and the solvent-accessible surface area method to calculate he apolar component. Calculation of binding free energy and per-residue decomposition of binding energies was achieved using the MmPbSaStat.py and MmPbSaDecomp.py scripts provided with g_MMPBSA.[27]

4. Supplementary Notes

Supplementary Note 1: Molecular Docking of Photoactive Probes

Highest ranked docking poses showed that **1a** and **20c** may be able to adopt a similar bound pose to **49c** with engagement of the catalytic dyad as well as key hydrogen bonds with the flap region – for **1a** this is inconsistent with *in vitro* activity, so docking may be insufficient to characterise unfavourable interactions of **1a** with the binding site. **20a** and **20b** both produced poses engaging just one of the catalytic aspartate residues, but also engaged Ser313 on the flap region – for **20b** this is inconsistent with the lower *in vitro* activity of this analogue. Compounds **1b** and **20d** failed to produce binding poses consistent with the MD derived bound pose of **49c** consistent with their *in vitro* activity. Interaction analyses are shown in Supplementary Figures 3-7.

5. Supplementary Figures

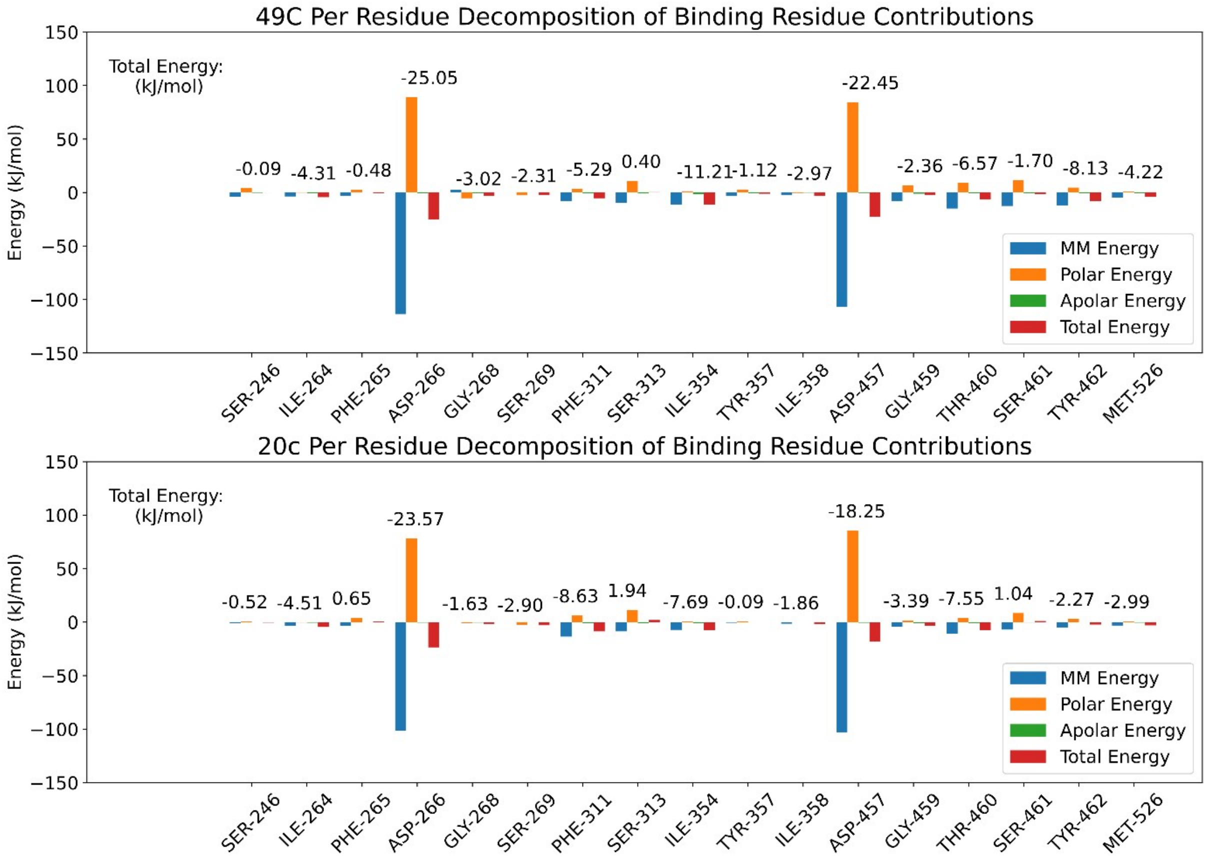

Supplementary Figure 1: Bar-plots showing binding site residue contributions to MM/PBSA binding energy for **49c** and compound **20c**. Values are derived from the first of three production MD runs for each. Total binding energy contributions are given for each residue.

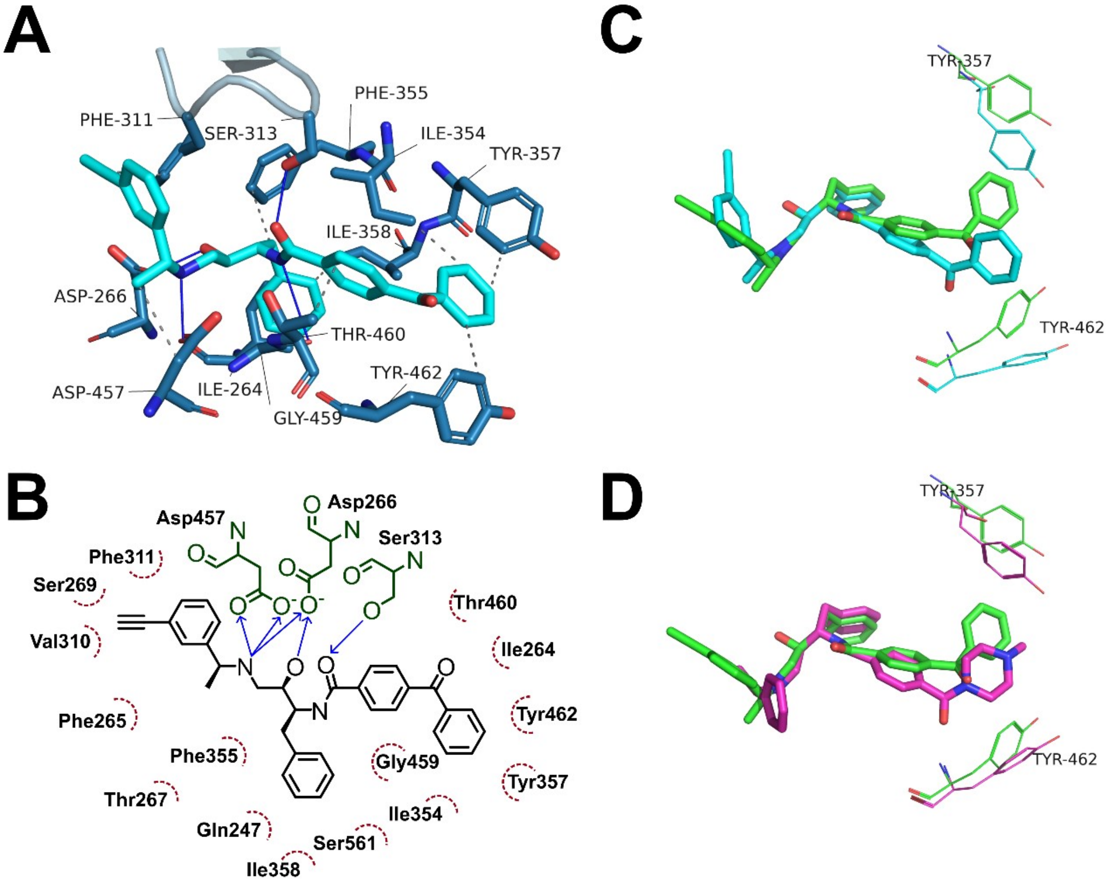

Supplementary Figure 2: A) Interaction analyses of end-state binding modes of **20c** from MD run 3 (cyan sticks) in PMX. Binding site residues are shown as blue sticks, the flap region is shown as a blue cartoon representation, hydrogen bonds are shown as blue lines, hydrophobic interactions are shown as dotted grey lines. Interactions were detected using the Protein Ligand Interaction Profiler tool. **B)** 2D representations of non-covalent interactions between **20c** and binding site residues. Binding site residues are shown in green, hydrophobic interacting residues are depicted by red, dotted arcs, hydrogen bond donors are depicted as blue arrows point from donor to acceptor. **C,D)** Alignment of the MD end state pose of **20c** from run 3 (green sticks) with the end state pose of **20c** from run 1 (cyan sticks, **C**) and the end state of **49c** from MD run 1 (magenta sticks, **D**). Pocket defining residues Tyr357, Ser461 and Tyr462 are shown as sticks. Note that in both cases the benzophenone carbonyl has shifted towards Tyr357 and is therefore unable to form hydrogen bonding interactions with Ser461 and Tyr462 backbone nitrogens.

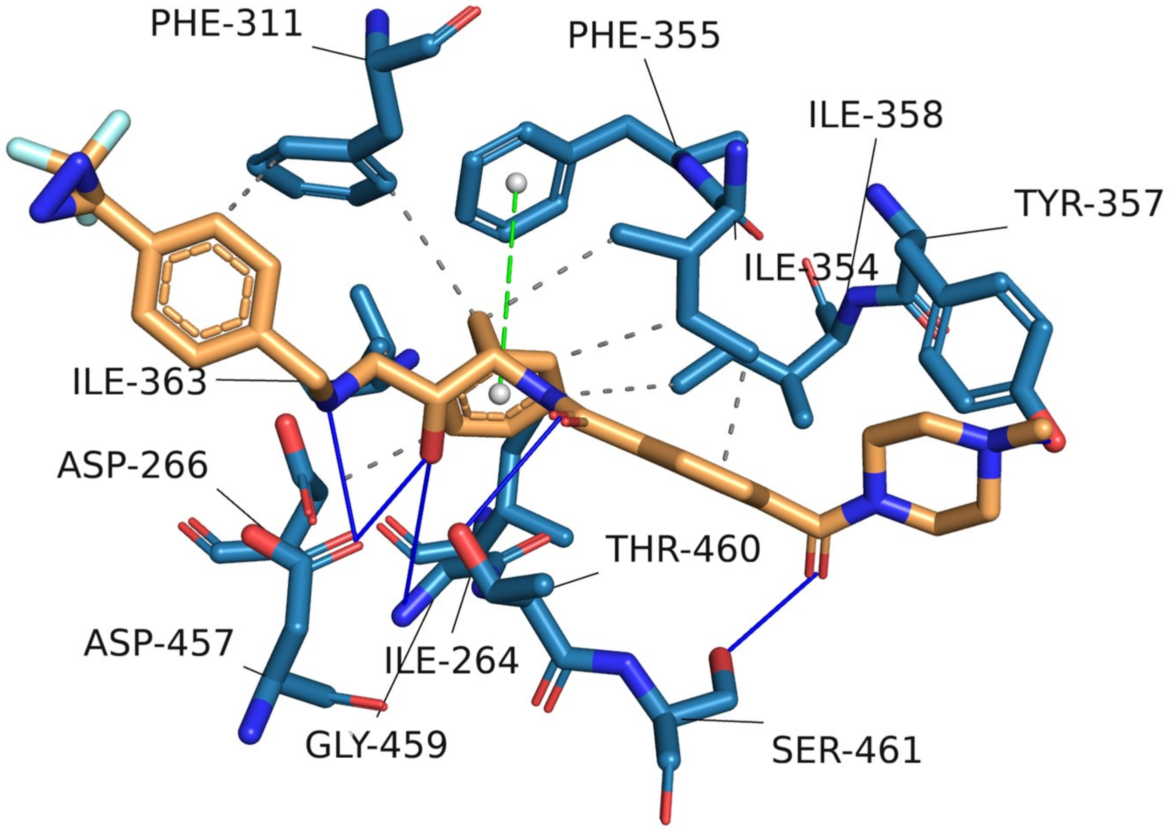

Supplementary Figure 3: Non-covalent interaction analysis of compound **1a** docked into PMX. Key: protein residues – blue sticks, **1a –** orange sticks, blue lines – hydrogen bonds, grey dotted lines – hydrophobic interactions, green dashed lines – pi-stacking interactions.

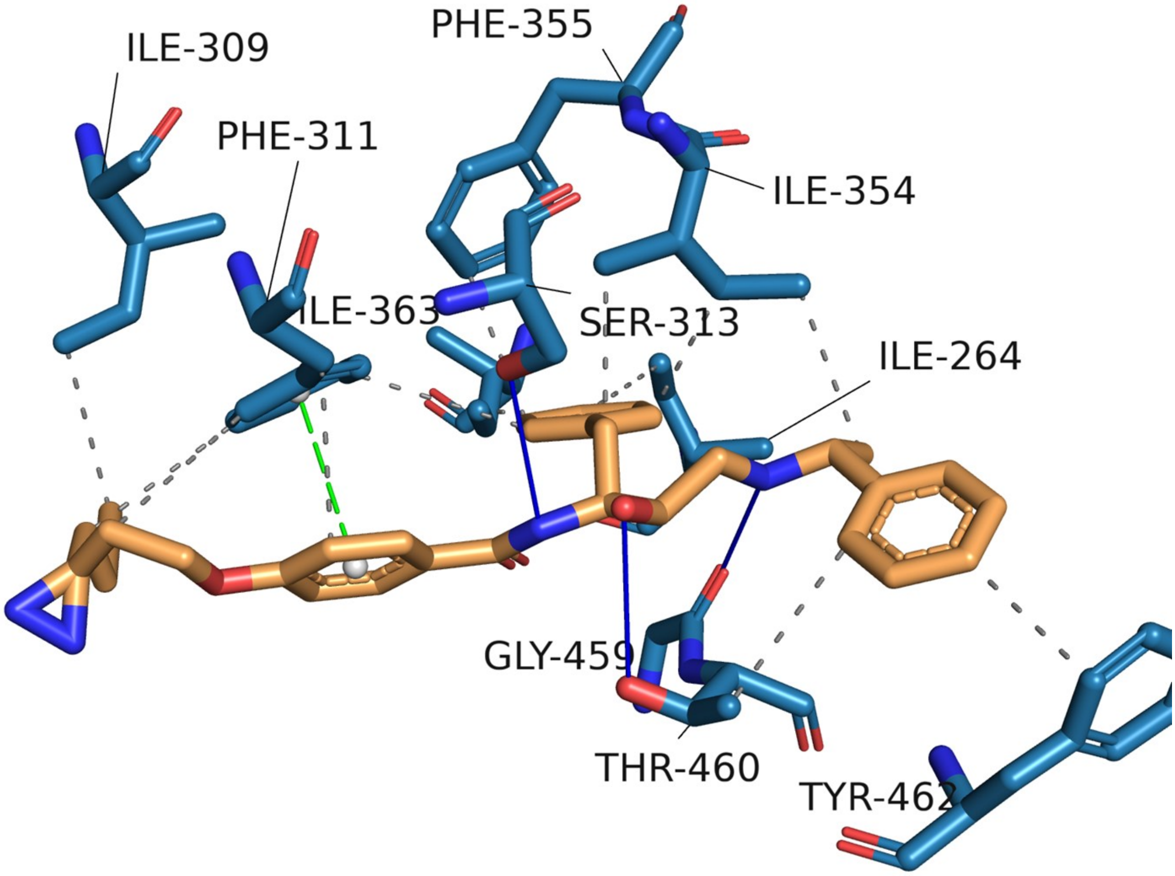

Supplementary Figure 4: Non-covalent interaction analysis of compound **1b** docked into PMX. Key: protein residues – blue sticks, **1b –** orange sticks, blue lines – hydrogen bonds, grey dotted lines – hydrophobic interactions, green dashed lines – pi-stacking interactions.

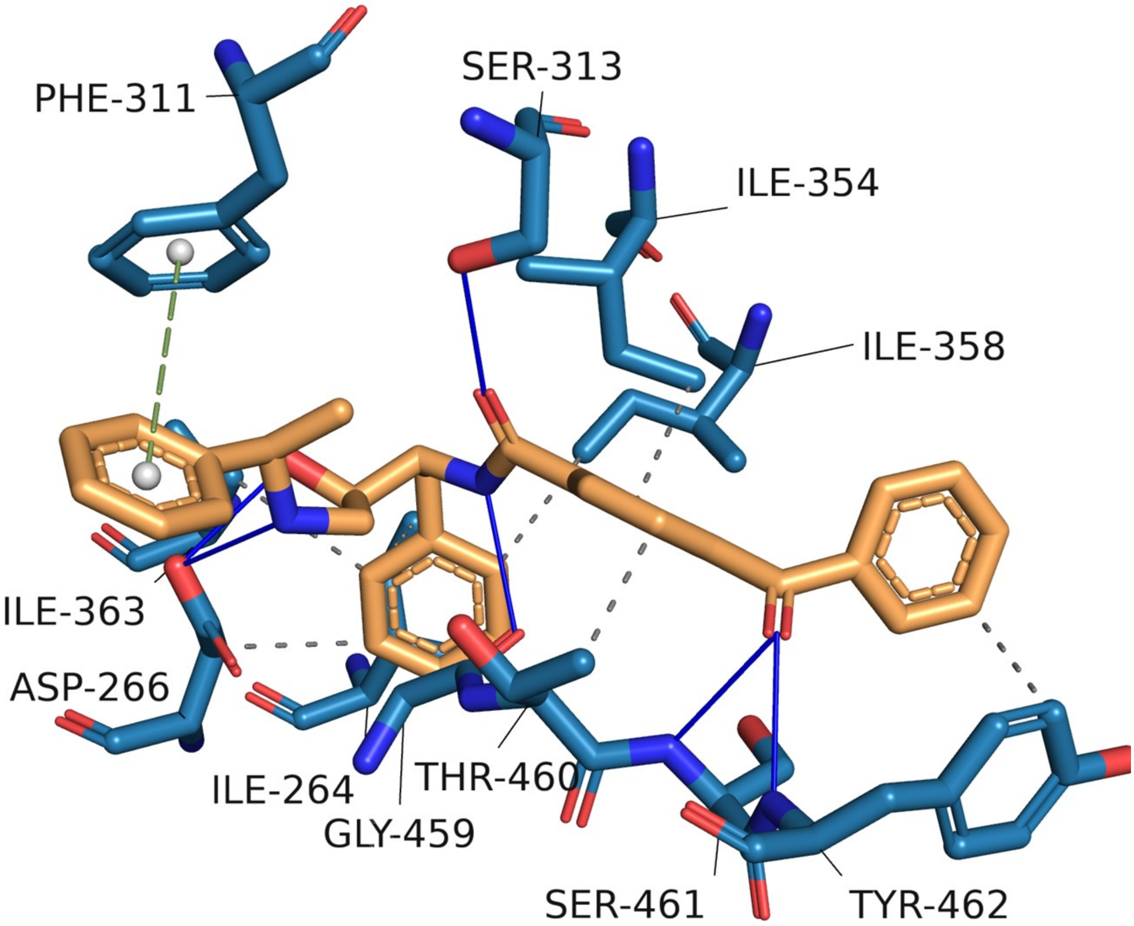

Supplementary Figure 5: Non-covalent interaction analysis of compound **20a** docked into PMX. Key: protein residues – blue sticks, **20a –** orange sticks, blue lines – hydrogen bonds, grey dotted lines – hydrophobic interactions, olive dashed lines – T-shaped pi-stacking interactions.

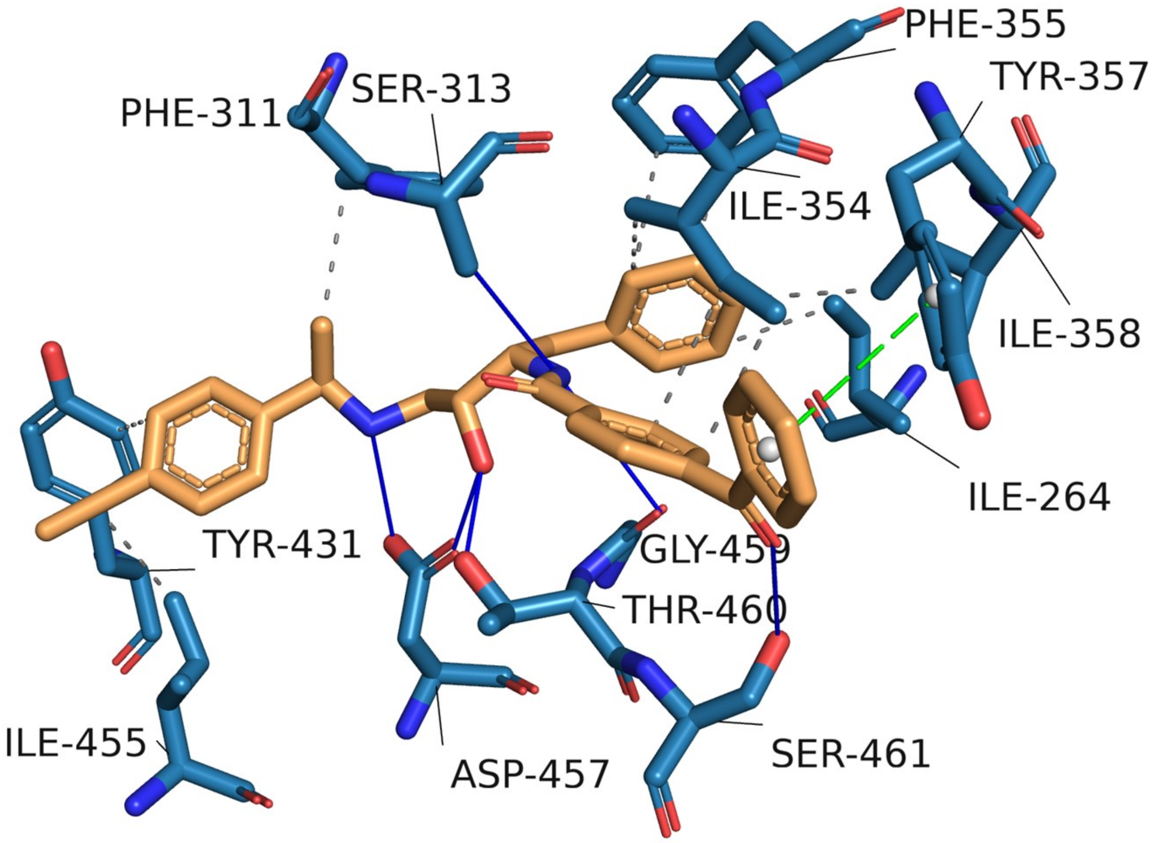

Supplementary Figure 6: Non-covalent interaction analysis of compound **20b** docked into PMX. Key: protein residues – blue sticks, **20b –** orange sticks, blue lines – hydrogen bonds, grey dotted lines – hydrophobic interactions, green dashed lines – pi-stacking interactions.

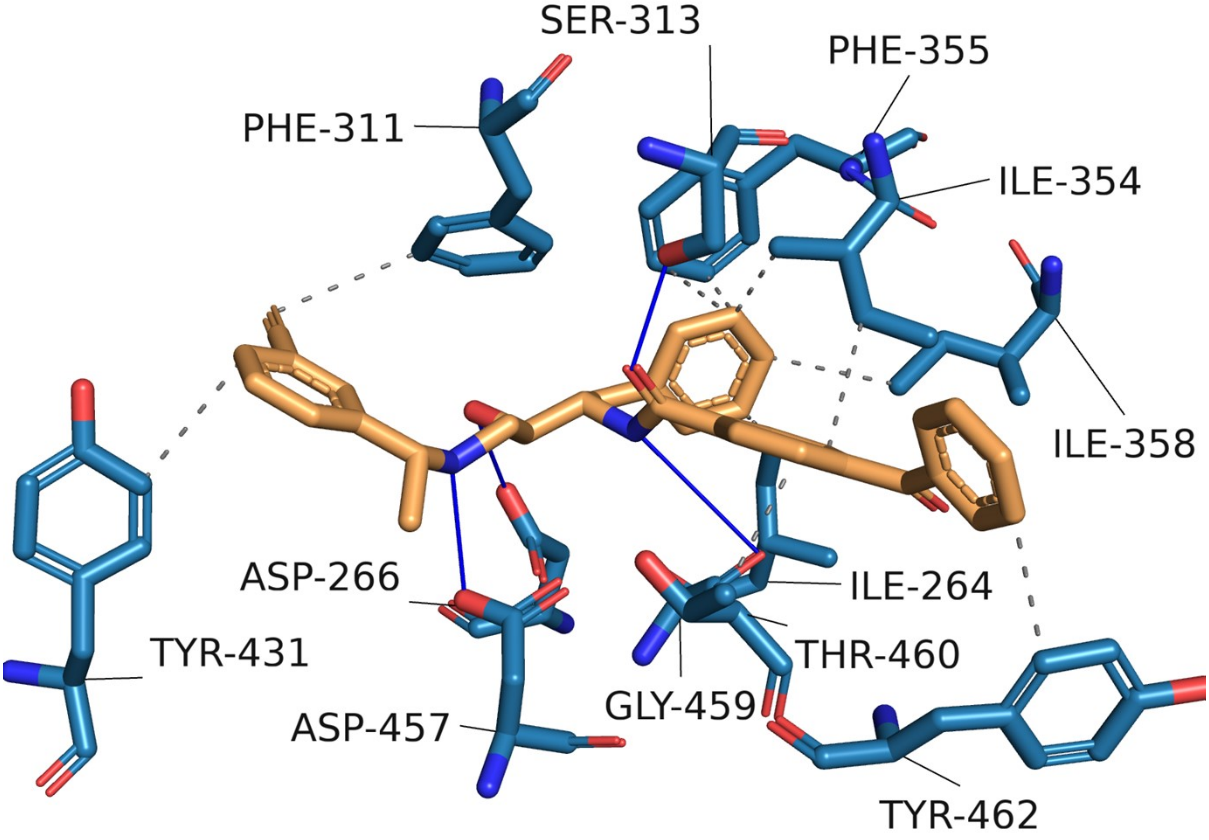

Supplementary Figure 7: Non-covalent interaction analysis of compound **20c** docked into PMX. Key: protein residues – blue sticks, **20c –** orange sticks, blue lines – hydrogen bonds, grey dotted lines – hydrophobic interactions.

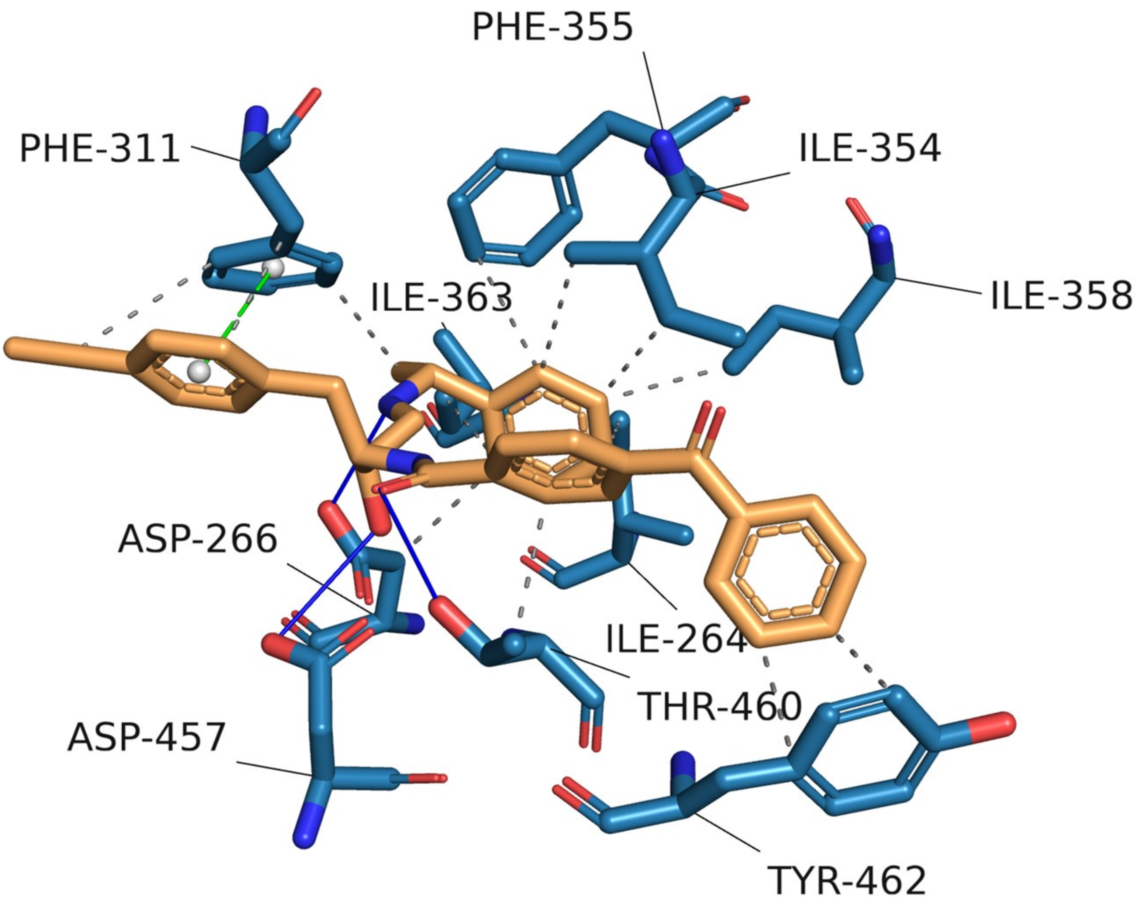

Supplementary Figure 8: Non-covalent interaction analysis of compound **20d** docked into PMX. Key: protein residues – blue sticks, **20d –** orange sticks, blue lines – hydrogen bonds, grey dotted lines – hydrophobic interactions, green dashed lines – pi-stacking interactions.

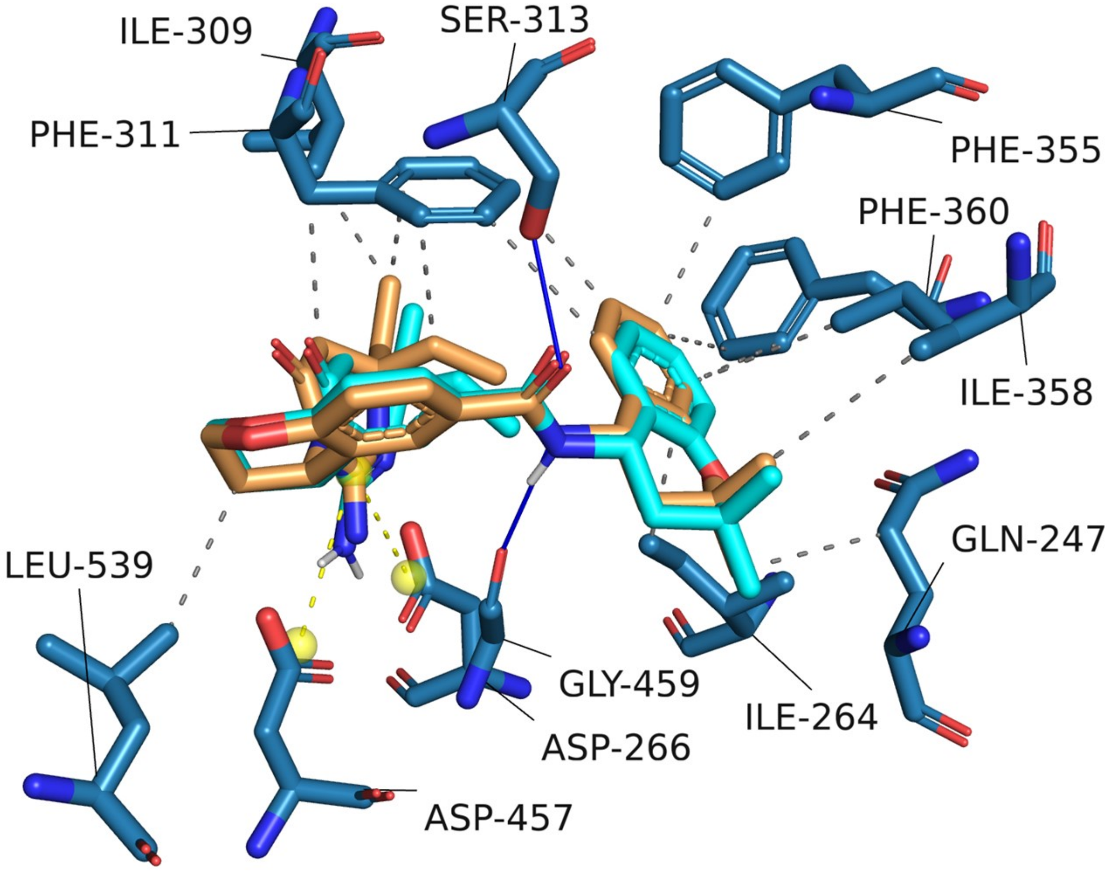

Supplementary Figure 9: Non-covalent interaction analysis of the reference ligand WM382 docked into PMX overlayed with the extracted conformation of WM382 observed in the crystal structure. Key: protein residues – blue sticks, **WM382** (docked) – orange sticks, **WM382** (co-crystallised) – cyan sticks, blue lines – hydrogen bonds, grey dotted lines – hydrophobic interactions, yellow dashed lines – salt bridge interactions, yellow-spheres – charge center.

6. Supplementary Tables

Supplementary Table 1: Summary of MM/PBSA calculated free energy components of ligand binding for the final 20 ns of each production MD run.

Compound	Run	ΔG_{VdW} (kJ/mol)	$\Delta G_{electrostatics}$ (kJ/mol)	ΔG_{polar} (kJ/mol)	ΔG_{apolar} (kJ/mol)	$\Delta G_{binding}$ (kJ/mol)
**49c**	1	-246.043 ± 18.10	-614.312 ±23.57	443.190 ± 18.19	-25.555 ±1.02	-442.720 ±16.82
	2	-233.123 ± 17.36	-625.889 ± 21.05	486.557 ±22.13	-23.394 ±1.01	-395.848 ±19.69
	3	-217.820 ±15.35	-574.444 ± 22.16	454.570 ±19.23	-23.765 ±1.27	-361.459 ±13.09
**20c**	1	-243.548 ±21.74	-559.904 ± 22.48	404.200 ±20.13	-26.984 ± 1.34	-426.236 ±21.36
	2	-237.627 ±24.17	-555.267 ±17.78	413.770 ±23.07	-26.517 ±1.45	-405.641 ±18.30
	3	-264.433 ±17.67	-552.295 ±21.58	414.332 ±23.63	-26.984 ±1.37	-429.381 ±19.32

Supplementary Table 2: Detailed analysis of interactions detected by clustering and stability of the interaction by presence in percentage of frames of each simulation. Important interactions are shown in bold

Residue	Interaction Type	**49c** Interactions (% present)			**20c** Interactions (% present)		
		Run_1	Run_2	Run_3	Run_1	Run_2	Run3
SER246	Hydrogen Bond	0.0	0.0	0.0	1.1	0.0	0.0
**ASP266**	**Hydrogen Bond**	**100.0**	**100.0**	**98.9**	**99.7**	**99.9**	**100.0**
SER269	Hydrogen Bond	75.3	0.0	34.4	0.0	0.0	4.9
GLY312	Hydrogen Bond	0.0	98.3	84.8	0.0	7.1	13.0
**SER313**	**Hydrogen Bond**	**88.1**	**12.8**	**34.7**	**60.2**	**83.0**	**79.7**
**ASP457**	**Hydrogen Bond**	0.0	0.0	3.7	1.7	0.4	3.5
GLY459	Hydrogen Bond	0.0	0.0	1.7	0.9	0.8	1.1
**THR460**	**Hydrogen Bond**	**97.6**	**85.6**	**94.1**	**61.1**	**60.1**	**57.7**
**SER461**	**Hydrogen Bond**	**98.4**	**99.2**	**95.0**	**84.1**	**84.4**	**79.2**
**TYR462**	**Hydrogen Bond**	**99.3**	**97.0**	**98.2**	**62.7**	**68.4**	**63.2**
LEU243	Hydrophobic	0.0	0.0	3.5	0.0	0.0	0.0
GLN247	Hydrophobic	0.0	0.5	23.2	0.0	0.0	5.6
PHE248	Hydrophobic	0.0	0.0	0.0	2.2	0.0	0.0
**ILE264**	**Hydrophobic**	**94.4**	**83.9**	**41.2**	**94.9**	**93.6**	**93.6**
ASP266	Hydrophobic	11.2	0.0	0.0	25.4	5.8	0.0
ILE309	Hydrophobic	0.0	0.0	7.8	6.2	0.0	3.4
**PHE311**	**Hydrophobic**	**75.5**	**95.1**	**58.8**	**66.5**	**70.5**	**74.1**
**ILE354**	**Hydrophobic**	**78.3**	**43.1**	**64.3**	**92.3**	**88.8**	**70.0**
**PHE355**	**Hydrophobic**	**61.4**	**0.8**	**0.0**	**33.7**	**64.8**	**73.4**
TYR357	Hydrophobic	0.0	0.0	0.0	46.0	32.0	14.2
**ILE358**	**Hydrophobic**	**81.4**	**43.6**	**64.2**	**52.0**	**63.1**	**74.7**
PHE360	Hydrophobic	0.0	51.8	22.5	0.0	0.0	0.0
ILE363	Hydrophobic	32.3	47.3	0.0	14.5	25.9	4.2
MET371	Hydrophobic	0.0	0.0	0.0	0.3	0.0	0.0
TYR431	Hydrophobic	0.0	0.0	0.0	6.9	0.0	1.1
ILE455	Hydrophobic	0.0	0.0	0.0	5.8	11.9	21.3
ASP457	Hydrophobic	0.0	0.0	3.3	23.1	19.9	36.0
**THR460**	**Hydrophobic**	**83.0**	**63.8**	**85.4**	**67.9**	**69.2**	**71.8**
TYR462	Hydrophobic	0.0	0.0	0.0	46.4	8.3	15.2
**LEU539**	**Hydrophobic**	**56.1**	**56.6**	**7.7**	**43.8**	**27.4**	**0.0**

7 References

1 C.-L. Ciana, R. Siegrist, H. Aissaoui, L. Marx, S. Racine, S. Meyer, C. Binkert, R. de Kanter, C. Fischli, S. Wittlin and C. Boss, *Bioorg. Med. Chem. Lett.*, 2013, **23**, 658–662.

2 K. Hayakawa, M. Yodo, S. Ohsuki and K. Kanematsu, *J. Am. Chem. Soc.*, 1984, **106**, 6735–6740.

3 M. Walko, E. Hewitt, S. E. Radford and A. J. Wilson, *RSC Adv.*, 2019, **9**, 7610–7614.

4 P. Kleiner, W. Heydenreuter, M. Stahl, V. S. Korotkov and S. A. Sieber, *Angew. Chemie Int. Ed.*, 2017, **56**, 1396–1401.

5 A. V. West, G. Muncipinto, H.-Y. Wu, A. C. Huang, M. T. Labenski, L. H. Jones and C. M. Woo, *J. Am. Chem. Soc.*, 2021, **143**, 6691–6700.

6 J. M. Sanderson, J. B. C. Findlay and C. W. G. Fishwick, *Tetrahedron*, 2005, **61**, 11244–11252.

7 WO2012078915A1, 2012, 1–152.

8 C. L. Oswald, T. Carrillo-Márquez, L. Caggiano and R. F. W. Jackson, *Tetrahedron*, 2008, **64**, 681–687.

9 W. Trager and J. B. Jensen, *Science (80-.).*, 1976, **193**, 673–675.

10 M. T. Makler, J. M. Ries, J. A. Williams, J. E. Bancroft, R. C. Piper, B. L. Gibbins and D. J. Hinrichs, *Am. J. Trop. Med. Hyg.*, 1993, **48**, 739–741.

11 N. Basilico, S. Parapini, A. Sparatore, S. Romeo, P. Misiano, L. Vivas, V. Yardley, S. Croft, A. Habluetzel, L. Lucantoni, L. Renia, B. Russell, R. Suwanarusk, F. Nosten, G. Dondio, C. Bigogno, D. Jabes and D. Taramelli, *Molecules*, 2017, **22**, 2102.

12 M. A. Lowe, A. Cardenas, J.-P. Valentin, Z. Zhu, J. Abendroth, J. L. Castro, R. Class, A. Delaunois, R. Fleurance, H. Gerets, V. Gryshkova, L. King, D. D. Lorimer, M. MacCoss, J. H. Rowley, M.-L. Rosseels, L. Royer, R. D. Taylor, M. Wong, O. Zaccheo, V. P. Chavan, G. A. Ghule, B. K. Tapkir, J. N. Burrows, M. Duffey, M. Rottmann, S. Wittlin, I. Angulo-Barturen, M. B. Jiménez-Díaz, J. Striepen, K. J. Fairhurst, T. Yeo, D. A. Fidock, A. F. Cowman, P. Favuzza, B. Crespo-Fernandez, F. J. Gamo, D. E. Goldberg, D. Soldati-Favre, B. Laleu and T. de Haro, *J. Med. Chem.*, 2022, **65**, 14121–14143.

13 A. N. Hodder, J. Christensen, S. Scally, T. Triglia, A. Ngo, R. W. Birkinshaw, B. Bailey, P. Favuzza, M. H. Dietrich, W.-H. Tham, P. E. Czabotar, K. Lowes, Z. Guo, N. Murgolo, M. de Lera Ruiz, J. A. McCauley, B. E. Sleebs, D. Olsen and A. F. Cowman, *Structure*, 2022, **30**, 947–961.

14 G. Jones, P. Willett, R. C. Glen, A. R. Leach and R. Taylor, *J. Mol. Biol.*, 1997, **267**, 727–748.

15 A. Kocak, I. Erol, M. Yildiz and H. Can, *J. Mol. Graph. Model.*, 2016, **70**, 226–235.

16 P. Kesari, A. Deshmukh, N. Pahelkar, A. B. Suryawanshi, I. Rathore, V. Mishra, J. H. Dupuis, H. Xiao, A. Gustchina, J. Abendroth, M. Labaied, R. Y. Yada, A. Wlodawer, T. E. Edwards, D. D. Lorimer and P. Bhaumik, *Protein Sci.*, 2022, **31**, 882–899.

17 S. Pronk, S. Páll, R. Schulz, P. Larsson, P. Bjelkmar, R. Apostolov, M. R. Shirts, J. C. Smith, P. M. Kasson, D. van der Spoel, B. Hess and E. Lindahl, *Bioinformatics*, 2013, **29**, 845–854.

18 K. Lindorff-Larsen, S. Piana, K. Palmo, P. Maragakis, J. L. Klepeis, R. O. Dror and D. E. Shaw, *Proteins Struct. Funct. Bioinforma.*, 2010, **78**, 1950–1958.

19 G. Bussi, D. Donadio and M. Parrinello, *J. Chem. Phys.*, 2007, **126**, 014101.

20 M. Parrinello and A. Rahman, *J. Appl. Phys.*, 1981, **52**, 7182–7190.

21 R. T. McGibbon, K. A. Beauchamp, M. P. Harrigan, C. Klein, J. M. Swails, C. X. Hernández, C. R. Schwantes, L.-P. Wang, T. J. Lane and V. S. Pande, *Biophys. J.*, 2015, **109**, 1528–1532.

22 P. Virtanen, R. Gommers, T. E. Oliphant, M. Haberland, T. Reddy, D. Cournapeau, E. Burovski, P. Peterson, W. Weckesser, J. Bright, S. J. van der Walt, M. Brett, J. Wilson, K. J. Millman, N. Mayorov, A. R. J. Nelson, E. Jones, R. Kern, E. Larson, C. J. Carey, İ. Polat, Y. Feng, E. W. Moore, J. VanderPlas, D. Laxalde, J. Perktold, R. Cimrman, I. Henriksen, E. A. Quintero, C. R. Harris, A. M. Archibald, A. H. Ribeiro, F. Pedregosa, P. van Mulbregt, A. Vijaykumar, A. Pietro Bardelli, A. Rothberg, A. Hilboll, A. Kloeckner, A. Scopatz, A. Lee, A. Rokem, C. N. Woods, C. Fulton, C. Masson, C. Häggström, C. Fitzgerald, D. A. Nicholson, D. R. Hagen, D. V Pasechnik, E. Olivetti, E. Martin, E. Wieser, F. Silva, F. Lenders, F. Wilhelm, G. Young, G. A. Price, G.-L.

Ingold, G. E. Allen, G. R. Lee, H. Audren, I. Probst, J. P. Dietrich, J. Silterra, J. T. Webber, J. Slavič, J. Nothman, J. Buchner, J. Kulick, J. L. Schönberger, J. V. de Miranda Cardoso, J. Reimer, J. Harrington, J. L. C. Rodríguez, J. Nunez-Iglesias, J. Kuczynski, K. Tritz, M. Thoma, M. Newville, M. Kümmerer, M. Bolingbroke, M. Tartre, M. Pak, N. J. Smith, N. Nowaczyk, N. Shebanov, O. Pavlyk, P. A. Brodtkorb, P. Lee, R. T. McGibbon, R. Feldbauer, S. Lewis, S. Tygier, S. Sievert, S. Vigna, S. Peterson, S. More, T. Pudlik, T. Oshima, T. J. Pingel, T. P. Robitaille, T. Spura, T. R. Jones, T. Cera, T. Leslie, T. Zito, T. Krauss, U. Upadhyay, Y. O. Halchenko, Y. Vázquez-Baeza and S. 1. . Contributors, *Nat. Methods*, 2020, **17**, 261–272.

23 S. Salentin, S. Schreiber, V. J. Haupt, M. F. Adasme and M. Schroeder, *Nucleic Acids Res.*, 2015, **43**, W443–W447.

24 E. N. Baker and R. E. Hubbard, *Prog. Biophys. Mol. Biol.*, 1984, **44**, 97–179.

25 N. Homeyer and H. Gohlke, *Mol. Inform.*, 2012, **31**, 114–122.

26 T. Hou, J. Wang, Y. Li and W. Wang, *J. Chem. Inf. Model.*, 2011, **51**, 69–82.

27 R. Kumari, R. Kumar and A. Lynn, *J. Chem. Inf. Model.*, 2014, **54**, 1951–1962.
